# Discovery of grey matter lesion-related immune genes for diagnostic prediction in multiple sclerosis

**DOI:** 10.7717/peerj.15299

**Published:** 2023-04-26

**Authors:** Peiyuan Zhao, Xihong Liu, Yunqian Wang, Xinyan Zhang, Han Wang, Xiaodan Du, Zhixin Du, Liping Yang, Junlin Hou

**Affiliations:** 1 School of Medicine, Henan University of Chinese Medicine, Zhengzhou, China; 2 The First Affiliated Hospital of Henan University of Chinese Medicine, Zhengzhou, China

**Keywords:** Multiple sclerosis, Grey matter lesion, Immune infiltration, Diagnosis

## Abstract

**Background:**

Multiple sclerosis (MS) is a chronic debilitating disease characterized by inflammatory demyelination of the central nervous system. Grey matter (GM) lesions have been shown to be closely related to MS motor deficits and cognitive impairment. In this study, GM lesion-related genes for diagnosis and immune status in MS were investigated.

**Methods:**

Gene Expression Omnibus (GEO) databases were utilized to analyze RNA-seq data for GM lesions in MS. Differentially expressed genes (DEGs) were identified. Weighted gene co-expression network analysis (WGCNA), least absolute shrinkage and selection operator (LASSO) algorithm and protein-protein interaction (PPI) network were used to screen related gene modules and candidate genes. The abundance of immune cell infiltration was analyzed by the CIBERSORT algorithm. Candidate genes with strong correlation with immune cell types were determined to be hub genes. A diagnosis model of nomogram was constructed based on the hub genes. Gene set enrichment analysis (GSEA) was performed to identify the biological functions of hub genes. Finally, an MS mouse model was induced to verify the expression levels of immune hub genes.

**Results:**

Nine genes were identified by WGCNA, LASSO regression and PPI network. The infiltration of immune cells was significantly different between the MS and control groups. Four genes were identified as GM lesion-related hub genes. A reliable prediction model was established by nomogram and verified by calibration, decision curve analysis and receiver operating characteristic curves. GSEA indicated that the hub genes were mainly enriched in cell adhesion molecules, cytokine-cytokine receptor interaction and the JAK-STAT signaling pathway, *etc*.

**Conclusions:**

TLR9, CCL5, CXCL8 and PDGFRB were identified as potential biomarkers for GM injury in MS. The effectively predicted diagnosis model will provide guidance for therapeutic intervention of MS.

## Introduction

Multiple sclerosis (MS) is a common type of neurodegenerative disease featuring inflammatory infiltration, demyelination and axonal damage (Tremlett & Marrie, 2021). Because diffuse demyelinating lesions appear in white-matter tracts, MS has historically been recognized as a white matter-dominated disease; however, recent neuroimaging and neuropathological studies have shown that grey matter (GM) damage exists in the early stages of MS and GM pathology has become a hallmark of progressive MS (Calabrese et al., 2015). During active GM inflammatory demyelination, widespread nonselective acute synaptic damage/loss is present. The synaptic loss has an impact on glutamatergic and GABAergic presynaptic terminals (Vercellino et al., 2022). In extensive GM damage of MS, in addition to focal demyelinating plaques, there is also diffuse neurodegeneration, manifested by an integral decrease in neuronal density, axonal damage and accumulation of oxidized phospholipids in neurons (Kawachi, 2014). Damaged neurons need to dissipate more energy to accommodate demyelination, thus creating an anoxia-like microenvironment. Such a microenvironment makes the surrounding surviving neurons more susceptible to degeneration, leading to atrophy of the GM and further exacerbating the severity of the disease (Eshaghi et al., 2018). Thus, GM pathology has a major impact on the irreversible physical and cognitive impairment of MS.

The plausible causes of the cortical neurodegeneration and GM damage in MS continue to be unclear, but it is presumed that a chronic pattern of inflammation is the major contributor. It has been suggested that the existence of pro-inflammatory infiltrates in the leptomeningeal or neighboring perivascular spaces probably results in the transfer of inflammatory agents to the GM (Gelfand, Cree & Hauser, 2017). For example, autopsies of patients with MS had revealed abnormal lymphoid structures with significant amounts of CD20^+^ B cells in inflamed meninges, and the accumulation of B cells was linked to sub mural demyelination injury (Reali et al., 2020). Additionally, although no signs of meningeal inflammation were found around some GM lesions, the active cortical demyelination damage suggested that this process may still be associated with immune events (Magliozzi, Reynolds & Calabrese, 2018). Gene expression profiles of GM lesions confirmed substantial pathological cellular alterations and indicated elevated levels of various signaling pathways involved in the immune response to inflammation (Magliozzi et al., 2019). It has been well established that CD4^+^ T cells may provoke an abnormal inflammatory response and secrete multiple dysregulated inflammatory factors causally related to the progression of MS (Zheng et al., 2019). Inflammatory factors enter the CNS and activate microglia and astrocytes. These two cells produce substantial chemokines and cytokines as well as recruit peripheral immune cells into the inflamed areas, leading to the progression of the disease. However, there is growing evidence that multiple lymphocyte subsets are involved in the complex and heterogeneous pathogenesis of MS, ranging from B cells, NK cells, and CD8^+^ T cells, demonstrating that these are promising targets for immunotherapy in MS (van Langelaar et al., 2020).

Therefore, we used bioinformatics strategies to analyze GM-related immune genes and immune infiltration in GM lesions, and integrated an MS diagnosis model. These genes were subsequently validated experimentally, which may provide potential targets for MS.

## Materials and Methods

### Data collection

In the Gene Expression Omnibus (GEO) database (http://www.ncbi.nlm.nih.gov/geo/), the keywords “multiple sclerosis”, “grey matter lesion”, “expression profiling by array” and “*Homo sapiens*” were used as screening criteria for the initial search. Datasets were then further filtered by applying the criteria below: (1) contained MS and normal group; (2) the samples were derived from the grey matter of the brain; and (3) the sample size was not less than 10. According to the above criteria, the GSE135511 dataset annotated by GPL6883 (Illumina HumanRef-8 v3.0 expression beadchip) and GSE131282 annotated by GPL10558 (HumanHT-12 V4.0 expression beadchip) were selected and downloaded. The GSE135511 dataset contained several subgroups including control GM, normal appearing GM, and GM lesion, *etc*. A total of ten samples of control GM tissues (GSM4013310 to GSM4013319) and ten samples of GM lesions (GSM4013300 to GSM4013309) were finally selected for subsequent study and analysis. GSE131282 was used as a validation dataset.

### Differentially expressed gene identification

The “R” software (R v4.1.3) was adopted for the analysis. By using the “limma” package (Ritchie et al., 2015), a filter of differentially expressed genes (DEGs) in GSE135511 was performed with cutoff values of adjusted *p*-value < 0.05 and |log2 fold change (FC)| ≥ 1. Volcano plots were created to show the differences in gene expression levels for the two groups. The clustering results are displayed by using the “pheatmap” package, which shows the top 20 up-regulated genes and the top 20 down-regulated genes.

### Functional enrichment analysis of DEGs

To reveal the biological functions of DEGs in GSE135511, enrichment analyses of the Gene Ontology (GO) and Kyoto Encyclopedia of Genes and Genomes (KEGG) were carried out using the R package “clusterProfiler” (Yu et al., 2012). By employing the “enrichplot” package, the results were visualized as bubble plots. The critical values of GO and KEGG were determined as *p* < 0.05.

### Weighted gene co-expression network analysis

WGCNA was performed on the gene expression profiles and clinical data in GSE135511 by using the “WGCNA” package to determine potential functioning modules (Langfelder & Horvath, 2008). All samples were clustered by average linkage and Pearson correlation value. A suitable soft threshold β was defined for the establishment of the scale-free network. The adjacency matrix was transformed into the topological overlap matrix (TOM). A hierarchical clustering tree was structured, and the branches of the clustering tree represented gene modules (minimum gene number of gene modules is 30). The correlation between the obtained modules and the clinical trait was analyzed, and the modules significantly correlated with the clinical trait were selected. A heatmap was also constructed to illustrate the correlation among different modules. The scatter plots were constructed to show the correlation between gene significance and module membership.

### Screening of immune-related genes

A list of immune-related genes was obtained from two databases: ImmPort (https://www.immport.org/) and InnateDB (https://www.innatedb.ca/). Modules most related to MS obtained by WGCNA were selected. The SangerBox (http://vip.sangerbox.com/) was used to draw a Venn diagram and show the intersection of immune-related genes and gene modules. The intersection genes were defined as immune-related mRNAs.

### Least absolute shrinkage and selection operator model construction

The least absolute shrinkage and selection operator (LASSO) method could obtain a refined model by employing a penalty function to reduce variable numbers. Applying the “glmnet” R package, LASSO regression analysis was performed, and penalty parameters were tuned by 10-fold cross-validation to filter immune-related candidate genes.

### Protein-protein interaction network analysis

The online STRING database (https://string-db.org) was used to identify potential interaction among the genes screened by LASSO. Then the result of STRING was uploaded to Cytoscape software. The cytoHubba plug-in was used to explore the hub genes in the PPI network. Top ten genes were generated using six common topological analysis methods in cytoHubba plug-in. The common hub genes were selected to perform subsequent analyses.

### Immune cell infiltration analysis

CIBERSORT is a widely used algorithm that uses linear support vector regression modeling to deconstruct the expression matrix of immune cell subtypes. To identify the immune characteristics of MS samples in GSE135511, the CIBERSORT was applied for assessing the proportions of 22 immune cell types (Newman et al., 2015). The relationship between immune cell subtypes and candidate genes were conducted by Pearson correlation analysis with “psych” and “corrplot” packages. The absolute value of correlation coefficient greater than 0.5 and *p*-value less than 0.05 were set as the cut-off.

### Nomogram model construction

In order to predict the onset of MS, a diagnostic nomogram model was developed by applying the “regplot” package. The accuracy and consistency of the nomogram model were assessed using calibration curves and decision curve analysis (DCA) curve. Receiver operating characteristic (ROC) curve analysis was performed with the “pROC” package. Diagnostic power of the hub gene in the GSE131282 dataset was checked with area under the curve (AUC).

### Gene set enrichment analysis

GSEA was carried out using the “clusterProfiler” package in order to identify biological functions of the hub genes in GSE135511. We chose “c2.cp.kegg.v7.5.1.symbols.gmt” as the reference gene list, |NES| > 1 and FDR < 0.25 indicated significant enrichment.

### Cuprizone-induced MS model

Male C57BL/6 mice (six-to-eight-week-old) were purchased from Zhejiang Vital River Laboratory Animal Technology Co., Ltd. (Zhejiang, China). Twenty mice were randomly divided into two groups (control and CPZ) of ten animals and each five mice were kept in one cage. All mice were housed in the specific pathogen-free facility under a 12-h light/dark cycle. The ambient temperature was 20–25 °C and the humidity was 50% ± 10%. For the induction of MS model, the CPZ mice were fed with 0.2% cuprizone chow for 5 weeks. The control mice were kept on a normal diet. Water was available *ad libitum*. Upon withdrawal of the cuprizone diet, the mice were euthanized by carbon dioxide asphyxiation, then the brain samples were collected. All experiments were approved by Experimental Animal Ethics Committee of Henan University of Chinese Medicine (Code: DWLL202103124). No excess experimental animals were used in this experiment.

### Luxol fast blue staining and immunohistochemical staining

Paraffin brain sections were deparaffinized, and LFB staining was applied to analyze demyelination according to standard protocol. For immunohistochemical (IHC) staining, sections were boiled in EDTA buffer for 20 min for antigen repair. The blocking solution was a PBS solution containing 5% BSA. Sections were then incubated with primary antibodies for MBP (1:50; Millipore), PDGFRB (1:1,000; Proteintech), TLR9 (1:500; Servicebio), and CCL5 (1:50; Huabio) overnight at 4 °C. After incubating the corresponding secondary antibody, images were obtained using Olympus BX53 microscopy. Three animals for each group were tested.

### Western-blotting analyses

Total proteins of cerebral cortex tissues of three animals for each group were extracted with RIPA buffer containing 1% PMSF and separated by 10% SDS-PAGE. Then, proteins were transferred to PVDF membranes. After blocking with 5% delipidated lipids for 1 h, the membranes were incubated overnight at 4 °C with the following primary antibodies: MBP (1:3,000; Millipore), PDGFRB (1:2,000; Proteintech), TLR9 (1:1,000; Servicebio), CCL5 (1:50; Huabio) and GAPDH (1:5,000; Proteintech). After incubation with HRP-conjugated secondary antibody, bands were visualized using ECL reagent on a Chemidoc detection system (Bio-Rad, Hercules, CA, USA). Three animals for each group were tested.

### Quantitative RT-PCR

Total RNA of cerebral cortex tissue was extracted using Trizol reagent (Ambion, Austin, TX, USA), followed by NanoDrop 2000 spectrophotometer (Thermo Fisher Scientific, Waltham, MA, USA) for quantitative analysis. Reverse transcription was performed using the Fastking RT Kit (Tiangen, Beijing, China). KAPA SYBR FAST (KAPA Biosystems, Wilmington, MA, USA) was used to detect the mRNA expression. The following PCR conditions were used: initial denaturation at 95 °C for 20 s, followed by 40 cycles of 95 °C for 3 s and 60 °C for 20 s. The primer sequences were as follows: TLR9 (forward: 5′-TATCCACCACCTGCACAACT-3′, reverse: 5′-TTCAGCTCCTCCAGTGTACG-3′), CCL5 (forward: 5′-TGCCCACGTCAAGGAGTATTTC-3′, reverse: 5′-AACCCACTTCTTCTCTGGGTTG-3′), PDGFRB (forward: 5′-AGCTACATGGCCCCTTATGA-3′, reverse: 5′-GGATCCCAAAAGACCAGACA-3′), GAPDH (forward: 5′-AGGTCGGTGTGAACGGATTTG-3′, reverse: 5′-GGGGTCGTTGATGGCAACA-3′). The results were gathered and analyzed by Quant Studio 7 Flex (Applied Biosystems, Foster City, CA, USA). The relative levels of target genes were calculated by using the 2^−ΔΔCT^ method, and an internal reference, GAPDH, was utilized. Four animals for each group were tested.

### Statistical analysis

Bioinformatic analyses were conducted using R software. Student’s *t* or Wilcoxon test was used to evaluate differences between two groups and was deemed significant at *p* < 0.05.

## Results

### Identification and enrichment analysis of DEGs

**Figure 1:**
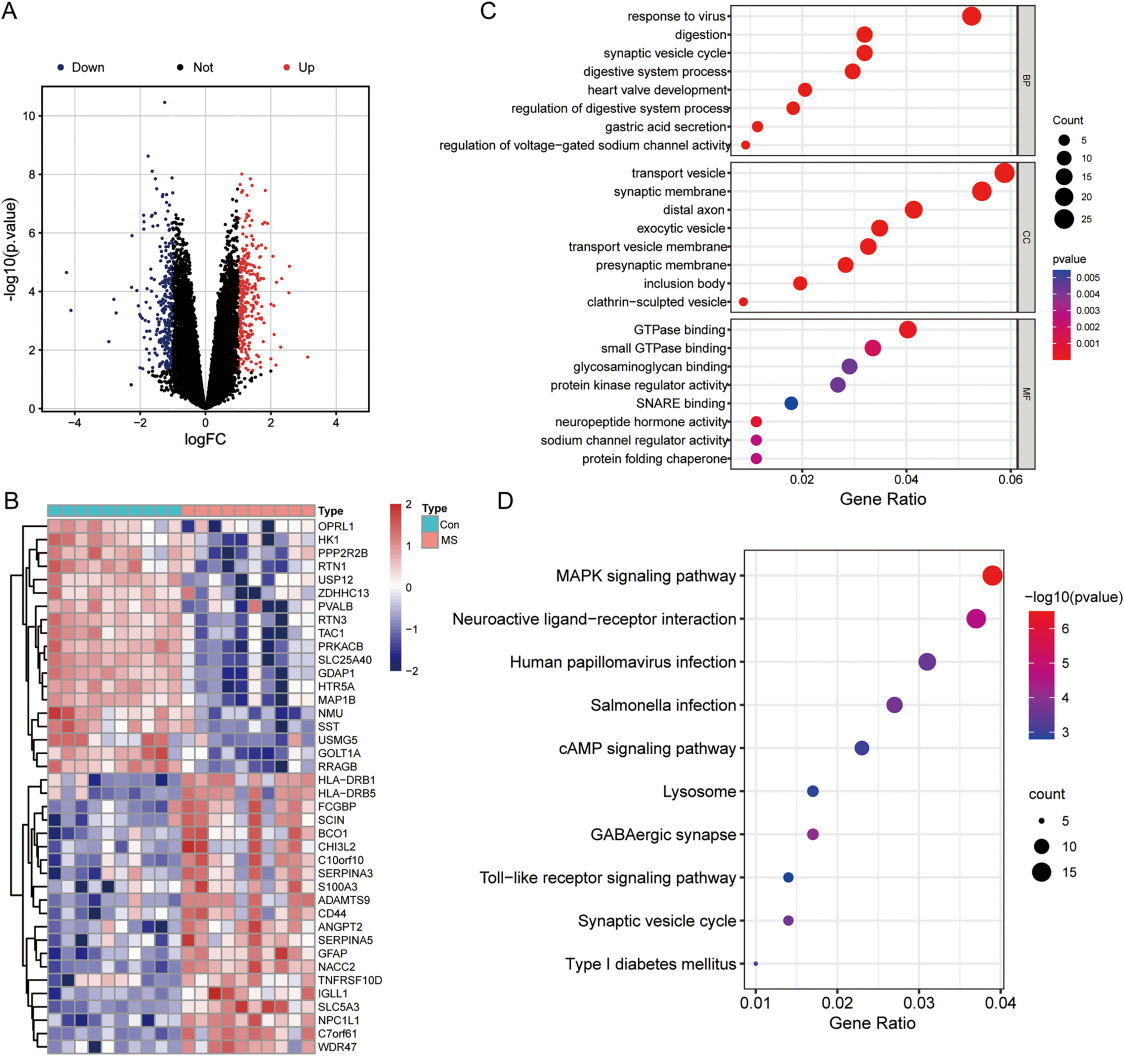
Differentially expressed genes (DEGs) analysis and GO, KEGG functional enrichment analysis. (A) Volcano plot of DEGs between normal and MS groups; red: up-regulation, blue: down-regulation. (B) Heatmap showing the top 20 up- and down-regulated genes in DEGs between normal and MS groups. (C) The enrichment results of biological process, cellular component, and molecular function by GO functional enrichment analysis. (D) Enriched functional categories of DEGs evaluated by KEGG pathway enrichment analysis.

The results of mRNA differential expression analysis showed a total of 502 DEGs, including 265 down-regulated genes and 237 up-regulated genes (Table S1). All these DEGs were displayed in the form of volcano plot (Fig. 1A). The heatmap depicts the top 20 up-and-down-regulated DEGs, with gene expression levels expressed in color, red for high expression, and blue for low expression (Fig. 1B). The results of the GO analysis showed that the enriched biological process (BP) terms were viral response, digestion, and synaptic vesicle cycles; the enriched cell composition (CC) terms were transport vesicles, synaptic membranes, and distal axon; the enriched molecular function (MF) terms were GTP enzyme binding, protein kinase regulator activity and sodium channel regulator activity (Fig. 1C). The top 10 enriched signaling pathways for the DEGs were determined by KEGG analysis (Fig. 1D), and DEGs identified were enriched in MAPK signaling pathways, neuroactive ligand-receptor interactions, and human tumor virus infection.

### Screening modules associated with GM lesions by WGCNA

**Figure 2:**
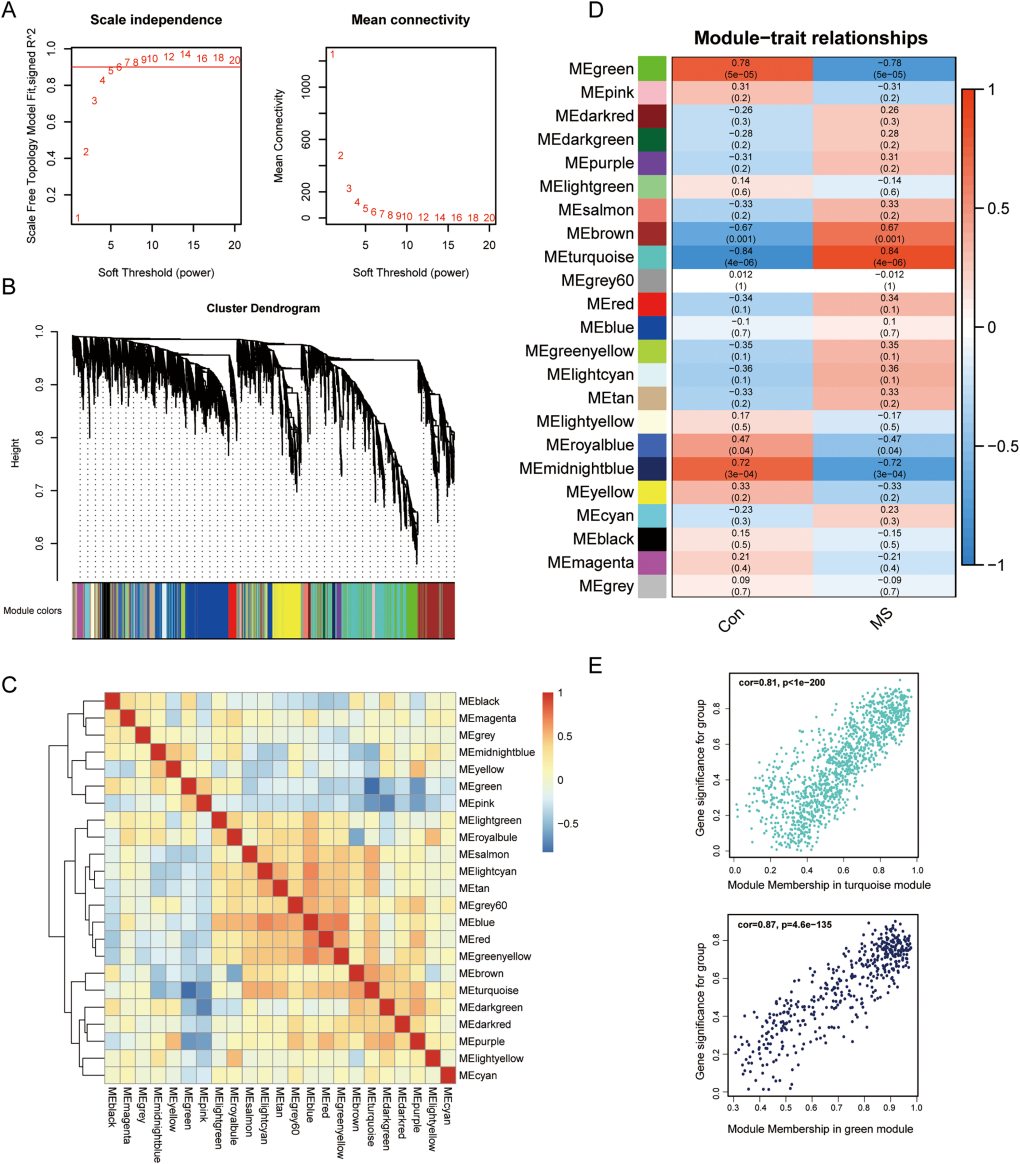
Screening for GM lesions related genes by WGCNA. (A) β = 6 (scale free R^2^ = 0.9) was chosen to construct a scale-free network. (B) Gene dendrogram obtained by average linkage hierarchical clustering, different colors represented the gene classification. (C) Correlation matrix be-tween different modules, red indicated high adjacency (positive correlation), and blue indicated low adjacency (negative correlation). (D) Correlation coefficients of the WGCNA modules between the normal and MS groups. (E) Scatter plots for correlations between gene significance and module membership in turquoise and green modules.

To uncover genes that significantly related to GM lesions, WGCNA was performed to identify potential functional modules. Genes with high expression correlations tend to be grouped into the same module, and each module is assigned a unique color, which implies that they are involved in a related biological pathway. Then, based on the scale-free R^2^ = 0.9 and high average connectivity, we set the soft threshold to 6 (Fig. 2A). The identified modules were shown under a clustering tree (Fig. 2B), and the reliability of module delineation was elucidated by correlation analysis between the modules, which showed that there was no substantial association between them (Fig. 2C). Correlations between modules and clinical traits were analyzed. The results showed that turquoise (|R| = 0.84, *p* = 4e−06) and green modules (|R| = 0.78, *p* = 5e−05) showed a strong correlation with MS, and therefore, these two modules were considered as key modules for further analysis. Correlation analysis of gene significance *vs*. module membership showed that they were highly correlated in the turquoise (R = 0.81, *p* < 1e−200) and green modules (R = 0.87, *p* = 4.6e−135) (Fig. 2E).

### Screening for immune-related genes

**Figure 3:**
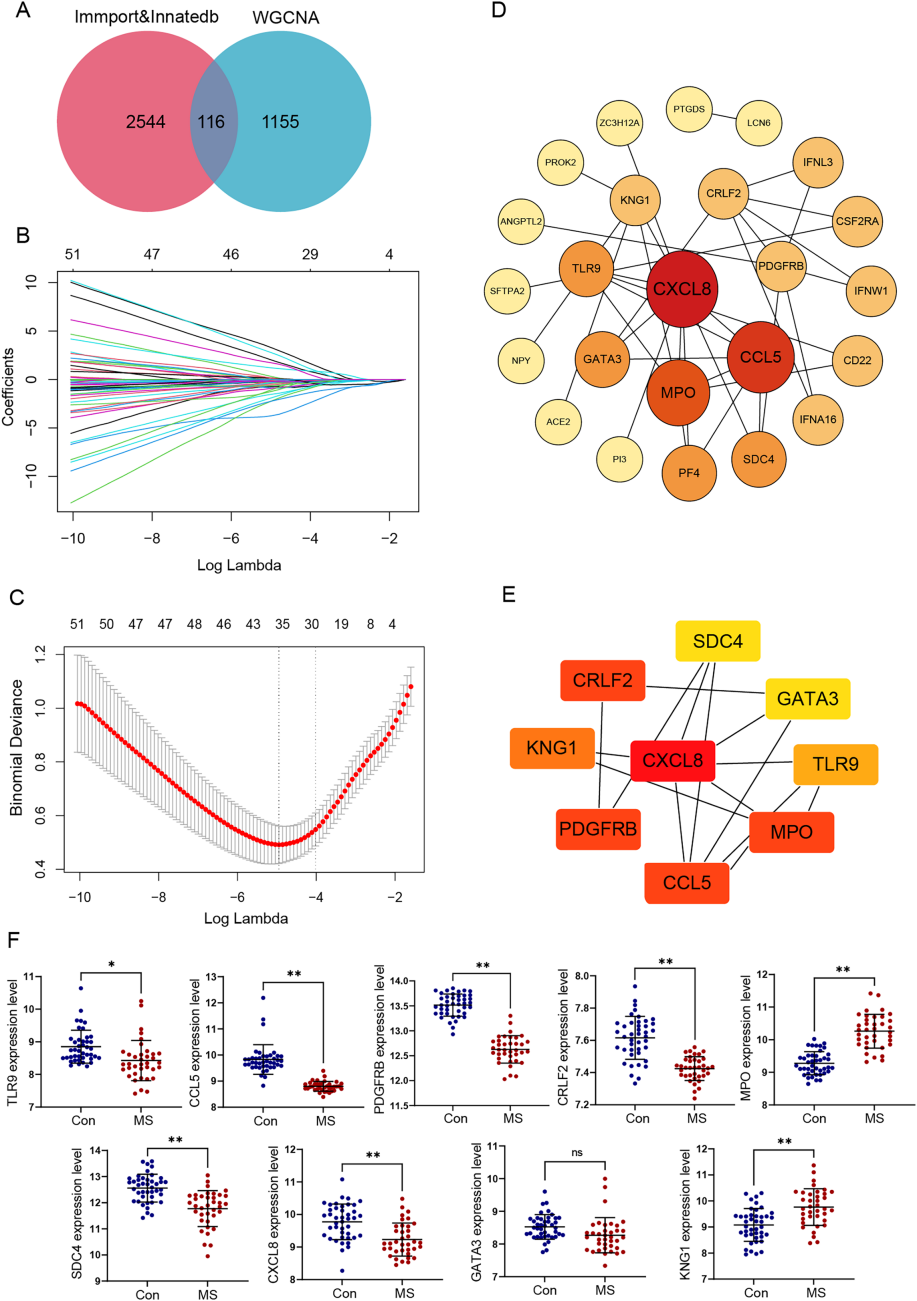
Identification and verification of GM lesions associated immune hub genes. (A) The Venn diagram shows intersection of immune-related genes and gene modules. (B) LASSO coefficient profiles of intersection genes. (C) Ten‐times cross-validation to select the optimal tuning parameter log (Lambda) in LASSO regression analysis. (D) Protein-protein interaction (PPI) of genes screened by LASSO. The color depth and shape size of the dots are positively correlated with degree. (E) Hub genes were explored by cytoHubba plug-in. (F) The different expression levels of candidate genes between normal and MS groups were validated in the GSE131282 dataset. *p < 0.05, **p < 0.01, and ns, no significance.

To explore immune-related gene modules, the PPI network was employed. We defined 2,660 immune-related genes from ImmPort and InnateDB (Table S2). In total 1,271 genes were identified after combining turquoise and green modules in WGCNA, and the intersection of these genes with immune genes was filtered (Fig. 3A).

We selected these 116 genes for LASSO analysis. With the increase of lambda value, the coefficient of some genes decreased to zero (Fig. 3B). Then tenfold cross-validation was carried out; when lambda = 0.018, the optimal model consisting of a total of 28 genes was developed (Fig. 3C).

PPI networks reflecting the association of the proteins encoded by LASSO screened genes were generated from the STRING database (Fig. 3D). Next, by taking the intersection of six algorithms of cytoHubba, a total of nine genes were selected, including CXCL8, CCL5, TLR9, MPO, KNG1, CRLF2, PDGFRB, SDC4 and GATA3 (Table 1). The significant module obtained from the PPI network using cytoHubba was shown in Fig. 3E.

**Table 1:** The top 10 hub genes rank in CytoHubba.

MCC	MNC	Degree	BottleNeck	Stress	EPC
CXCL8	CXCL8	CXCL8	CXCL8	CXCL8	CXCL8
CCL5	CCL5	CCL5	GATA3	GATA3	CCL5
MPO	MPO	MPO	CRLF2	CRLF2	MPO
TLR9	TLR9	PDGFRB	KNG1	SDC4	TLR9
CRLF2	PDGFRB	CRLF2	PDGFRB	PDGFRB	GATA3
PDGFRB	CRLF2	KNG1	SDC4	KNG1	PF4
PF4	PF4	TLR9	CCL5	CCL5	SDC4
KNG1	GATA3	PF4	MPO	MPO	PDGFRB
GATA3	SDC4	GATA3	CSF2RA	CSF2RA	CRLF2
SDC4	KNG1	SDC4	TLR9	TLR9	KNG1

Additionally, the expression values of these nine genes were verified using the data of 42 healthy control and 37 GM lesions tissue samples in the GSE131282 dataset, and eight of them (CXCL8, CCL5, TLR9, MPO, KNG1, CRLF2, PDGFRB and SDC4) were differentially expressed and exploited for further study (Fig. 3F).

### Correlation analysis of immune-related genes and immune cells

**Figure 4:**
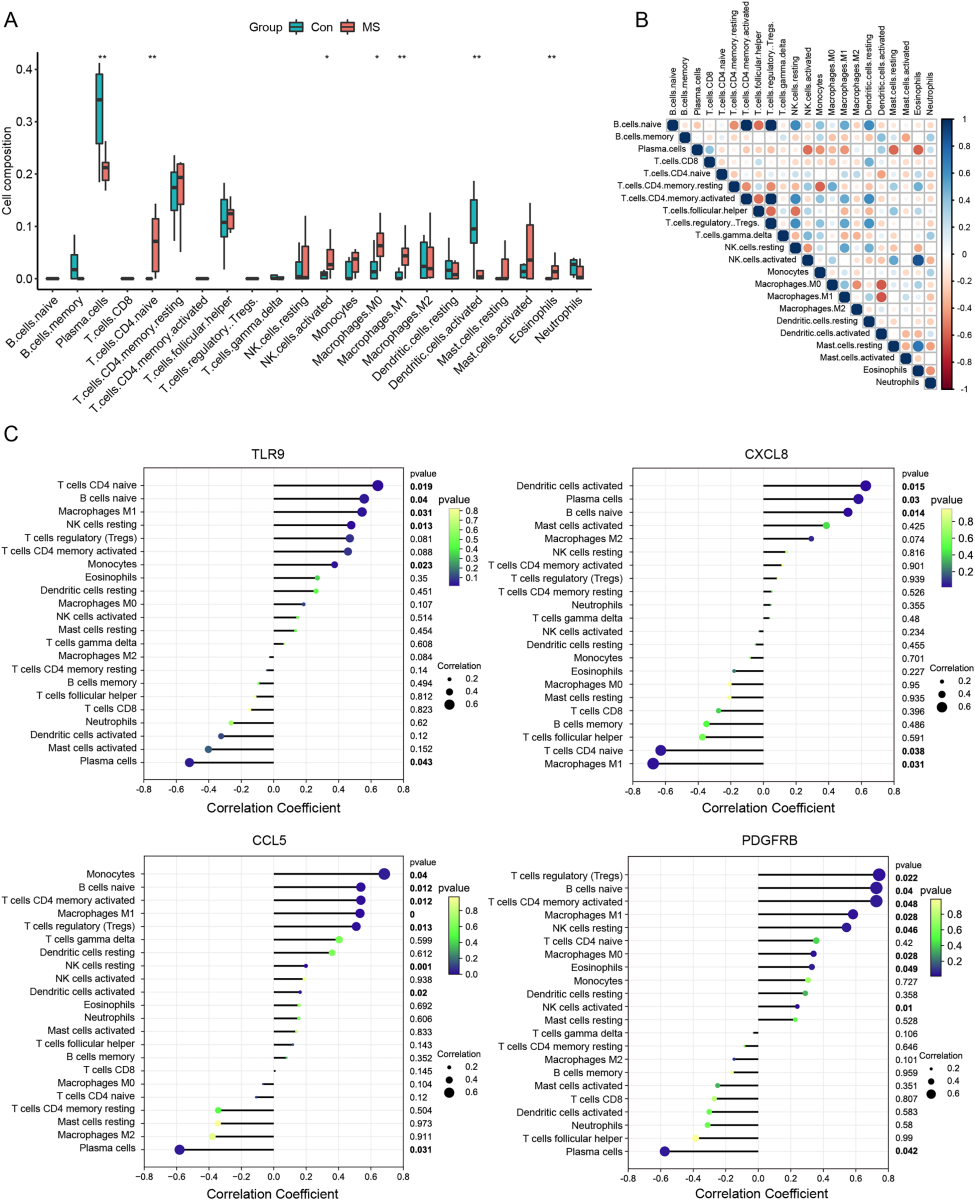
Immune cell infiltration analysis. (A) The relative abundance of 22 immune cell types between normal and MS groups. (B) The correlation matrix between immune cells. Red indicates positive correlation; blue indicates negative correlation. (C) Correlations between hub genes (TLR9, CXCL8, CCL5 and PDGFRB) and the infiltration levels. *p < 0.05, **p < 0.01.

Meanwhile, we calculated the infiltration of immune cells in MS samples, and the results showed that proportions of seven types of immune cells were significantly different between the two groups (Fig. 4A). There was an obvious increase in the proportion of naive CD4^+^ T cells, activated NK cells, M0 macrophages, M1 macrophages and eosinophils in MS group relative to that in normal group, while plasma cells and activated dendritic displayed the opposite proportion. The correlation of 22 types of immune cells was calculated (Fig. 4B). Naive B cells were significantly negatively correlated with CD4 memory resting T cells (R = −0.47, *p* = 0.040), but significantly positively correlated with resting NK cells (R = 0.64, *p* = 0.003), M1 macrophages (R = 0.51, *p* = 0.022) and resting dendritic cells (R = 0.62, *p* = 0.003). Plasma cells were significantly negatively correlated with activated NK cells (R = −0.56, *p* = 0.011), monocytes (R = −0.45, *p* = 0.044), M1 macrophages (R = −0.44, *p* = 0.050) and resting mast cells (R = −0.57, *p* = 0.008). CD8 T cells were significantly positively correlated with resting dendritic cells (R = 0.49, *p* = 0.029). CD4 memory resting T cells were significantly negatively correlated with CD4 memory activated T cells (R = −0.47, *p* = 0.038), regulatory Tregs T cells (R = −0.48, *p* = 0.032) and monocytes (R = −0.58, *p* = 0.007), but significantly positively correlated with M0 macrophages (R = 0.49, *p* = 0.027) and eosinophils (R = 0.48, *p* = 0.030). CD4 memory activated T cells were significantly negatively correlated with follicular helper T cells (R = −0.55, *p* = 0.011), but significantly positively correlated with resting NK cells (R = 0.64, *p* = 0.003), M1 macrophages (R = 0.51, *p* = 0.022) and resting dendritic cells (R = 0.62, *p* = 0.003). Follicular helper T cells were significantly negatively correlated with regulatory Tregs T cells (R = −0.57, *p* = 0.009) and resting NK cells (R = −0.54, *p* = 0.013). Regulatory Tregs T cells were significantly positively correlated with resting NK cells (R = 0.61, *p* = 0.004), M1 macrophages (R = 0.53, *p* = 0.017) and resting dendritic cells (R = 0.60, *p* = 0.005). Resting NK cells were significantly negatively correlated with activated NK cells (R = −0.44, *p* = 0.050), but significantly positively correlated with M1 macrophages (R = 0.53, *p* = 0.016) and resting dendritic cells (R = 0.48, *p* = 0.031). Activated NK cells were significantly positively correlated with resting mast cells (R = 0.51, *p* = 0.020). M0 macrophages were significantly negatively correlated with M2 macrophages (R = −0.46, *p* = 0.041) and activated dendritic cells (R = −0.61, *p* = 0.004). Resting mast cells were significantly positively correlated with eosinophils (R = 0.73, *p* < 0.001).

We further conducted correlation analysis to determine the association between the immune cells and the seven candidate genes (Fig. 4C). The results showed that CXCL8 displayed the most negative correlation with M1 macrophages (R = −0.67, *p* = 0.03) and the most positive correlation with activated dendritic cells (R = −0.62, *p* = 0.01), CCL5 displayed the most positive correlation with monocytes (R = 0.68, *p* = 0.03) and the most negative correlation with plasma cells (R = −.58, *p* = 0.03). TLR9 showed the most positive correlation with naive CD4^+^ T cells (R = 0.64, *p* = 0.01) and the most negative correlation with plasma cells (R = −0.51, *p* = 0.04), PDGFRB showed the most positive correlation with regulatory T cells (R = 0.74, *p* = 0.02) and the most negative correlation with plasma cells (R = −0.57, *p* = 0.04). Statistically significant correlations were not observed between the other four candidate genes (MPO, KNG1, CRLF2 and SDC4) and the 22 types of immune cells. Therefore, four genes (CXCL8, CCL5, TLR9 and PDGFRB) were finally determined to be immune-related hub genes.

### Diagnostic model construction

**Figure 5:**
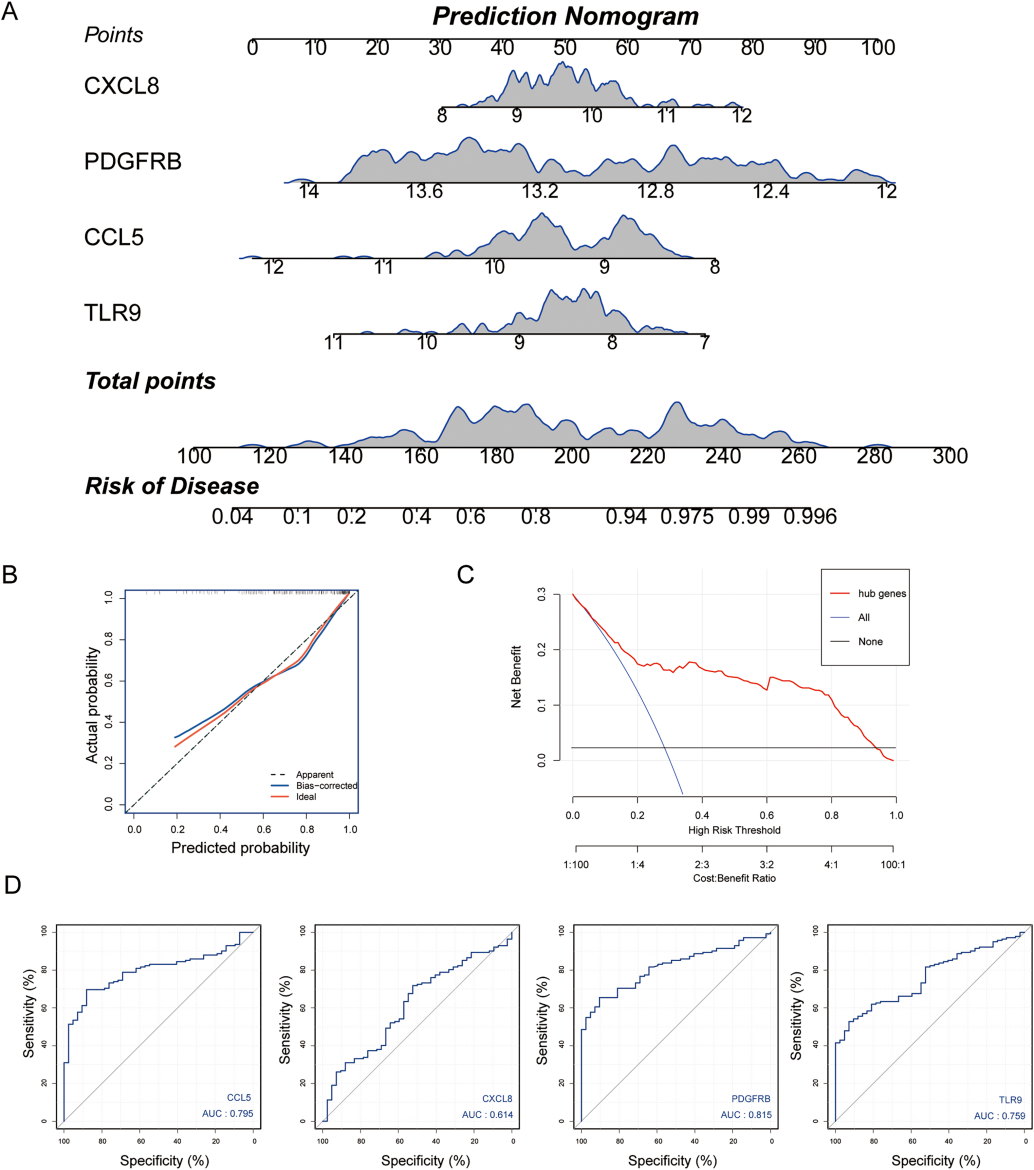
Nomogram model construction for MS diagnosis. (A) Nomogram to predict MS risk. (B) Calibration curve evaluation for the diagnostic potential of the nomogram model. (C) The decision curve analysis (DCA) curve for assessment of the performance of nomogram. (D) ROC curve to evaluate prediction accuracy of the four hub genes in GSE131282.

Subsequently, we established a diagnosis nomogram model based on gene signatures (Fig. 5A). In order to detect the performance of the nomogram, we built a calibration curve for visualization (Fig. 5B); the nomogram prediction model displayed a C-index of 0.854 which revealed high consistency between the actual and predicted survival rates. According to the DCA curve (Fig. 5C), the nomogram prediction model has good performance in predicting the incidence of MS. Subsequently, we conducted ROC curve analysis in the third-party dataset GSE131282. The AUC values of hub genes were CCL5: 0.795, CXCL8: 0.614, PDGFRB: 0.815, TLR9: 0.759 (Fig. 5D). Hence, the nomogram based on a four-gene signature is a good model for MS diagnosis.

### Analysis of GSEA pathways of immune hub genes in MS

**Figure 6:**
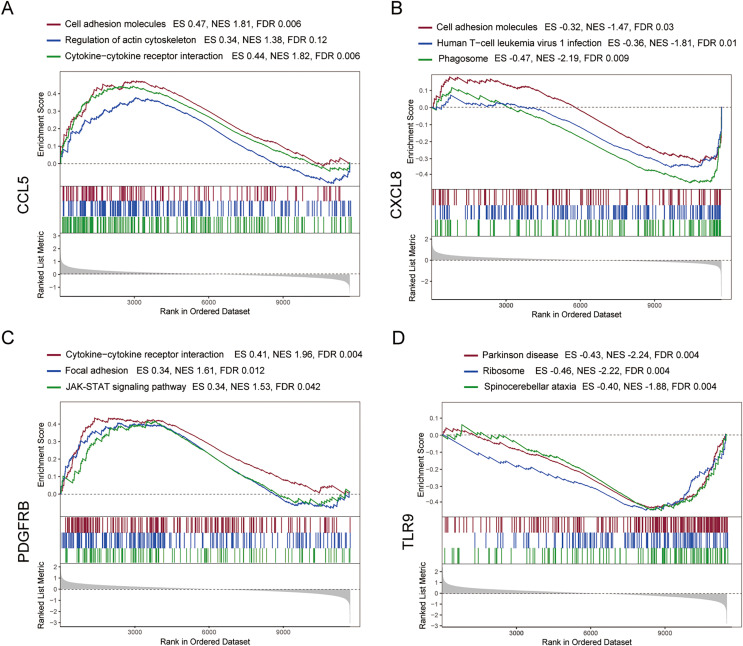
GSEA analysis of the immune-related hub genes. (A–D) Gene set enrichment analysis for CCL5, CXCL8, PDGFRB and TLR9.

**Figure 7:**
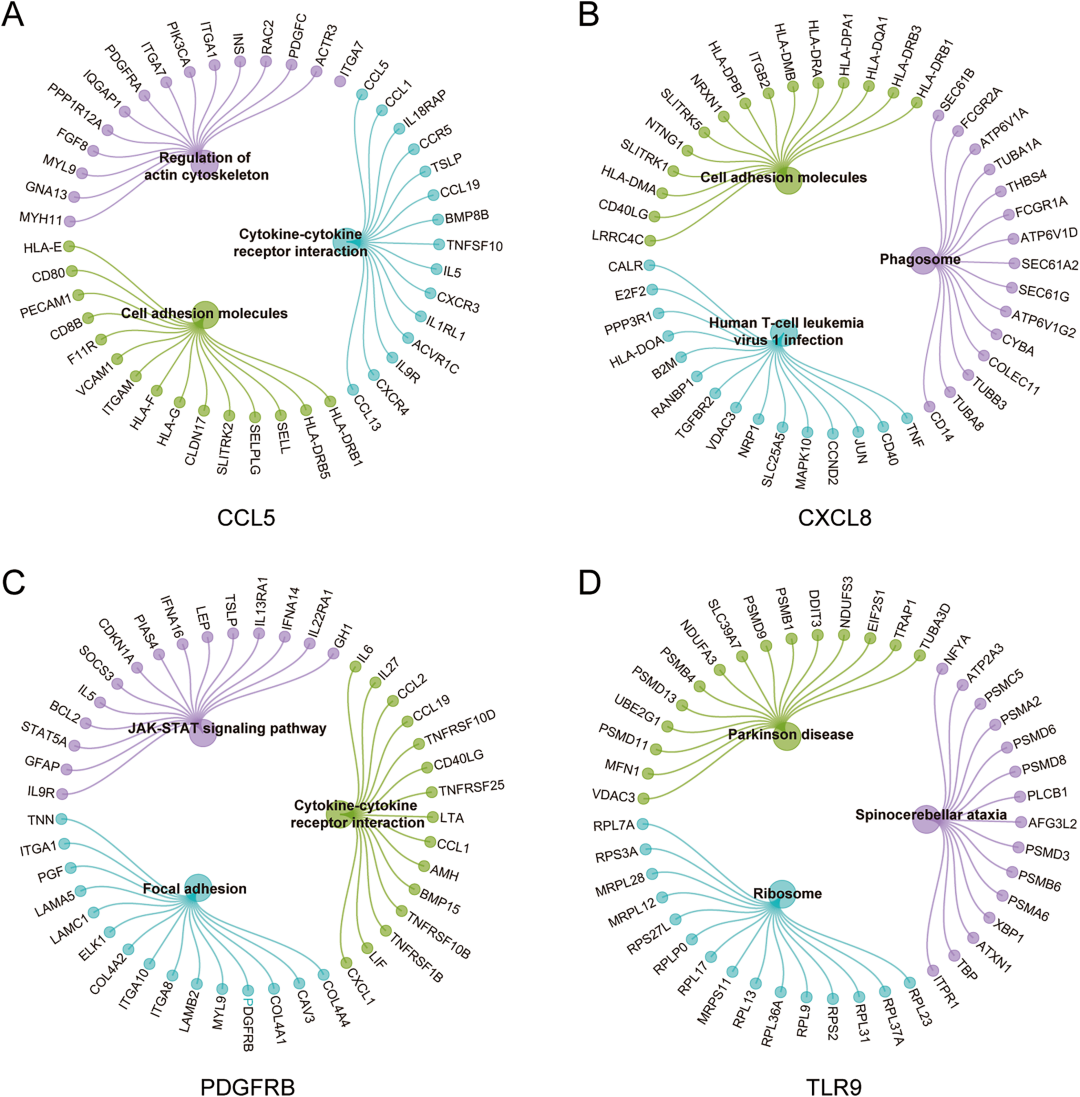
Genes significantly enriched in immune hub genes-related pathways. (A–D) GSEA-related flower plot of CCL5, CXCL8, PDGFRB and TLR9.

To further explore the hub gene-related signaling pathway, the GSEA method was used. GSEA enrichment analysis showed a dominant enrichment of CCL5 in the pathways of adhesion molecules, regulation of actin cytoskeleton and cytokine-cytokine receptor interactions (Fig. 6A). CXCL8 was prominently enriched in the pathways of cell adhesion molecules, human T-cell leukemia virus 1 infection and Phagosome (Fig. 6B). PDGFRB exhibited a significant enrichment in the pathways of cytokine-cytokine receptor interaction, focal adhesion and JAK-STAT signaling pathway (Fig. 6C). TLR9 was significantly enriched in the pathways of Parkinson disease, ribosome, and spinocerebellar ataxia (Fig. 6D). Genes significantly enriched in hub gene-related pathways were shown in Fig. 7.

### Verification of immune hub genes in MS mouse model

**Figure 8:**
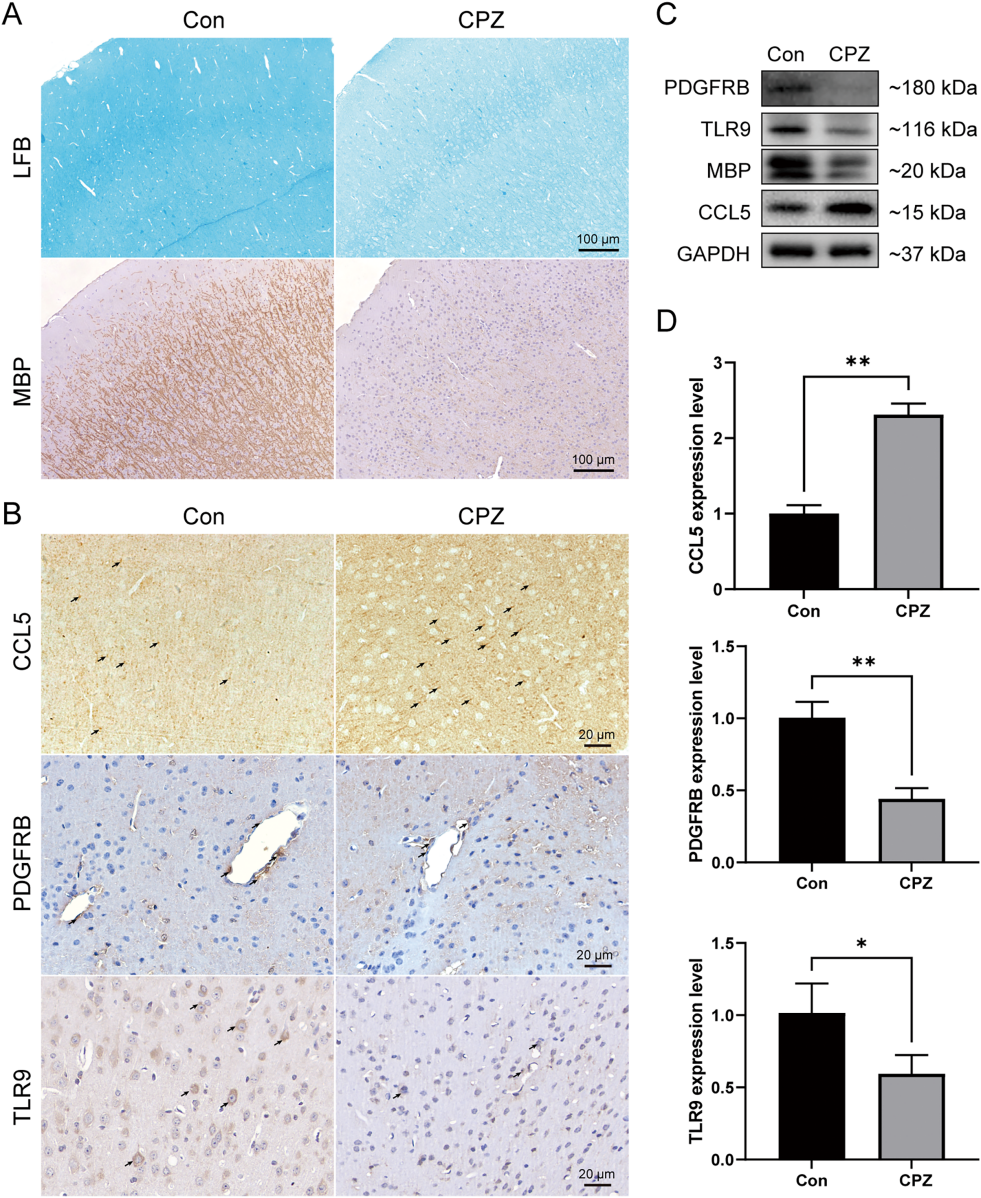
Expression levels of CCL5, PDGFRB and TLR9 protein and mRNA in cuprizone-induced MS models. (A) Induction of demyelination in the cerebral cortex of CPZ-fed mice. Luxol Fast Blue and immunostaining for MBP on brain sections of CPZ and control groups (*n* = 3 for each group). (B) Immunostaining of CPZ and control groups, labelled with CCL5, PDGFRB and TLR9 (indicated by arrows, *n* = 3 for each group). The protein (C) and mRNA (D) levels of CCL5, PDGFRB and TLR9 in the cerebral cortex tissues of CPZ and control mice by western blot (*n* = 3 for each group) and RT-qPCR (*n* = 4 for each group). Data were expressed as mean ± SD (*p < 0.05, **p < 0.01).

To analyze the myelin loss in the cerebral cortex of CPZ-fed mice, LFB staining for myelin sheath and IHC staining for MBP (a marker of mature myelin) were performed (Fig. 8A). We observed a strong myelin loss in mice cortical tissues after 5 weeks of treatment with CPZ. We further detected the protein level of MBP in cortical tissues using western blot, and in line with the IHC result, MBP level was significantly downregulated in the CPZ group, indicating that CPZ successfully induced cortical demyelination. As rodents lack the CXCL8 gene, we verified the protein and mRNA expressions of TLR9, PDGFRB and CCL5 in cuprizone-induced MS mice by IHC, western blot and RT-qPCR (Figs. 8B–8D). The results showed that compared with control group, the protein and mRNA expressions of TLR9 and PDGFRB were decreased, while the protein and mRNA expression of CCL5 was increased in CPZ group.

## Discussion

As an inflammatory-mediated demyelinating disease, MS is considered to be a primary contributor to neurological disability, most notably in young adults. What makes the pathogenesis of multiple sclerosis more difficult to elucidate is the variety of lesion types that patients exhibit at different clinical stages (Lassmann, 2018). Studies have confirmed that GM injury is more relevant to cognitive impairment, depression, and physical disability in MS (Hnilicova et al., 2020). Inflammation of the soft meninges is recognized as one of the main traits of secondary progressive MS with the possibility of causing widespread cortical lesions associated with progressive disease. Moreover, inflammation in the soft meninges correlates with neuronal damage in the GM, therefore it is hypothesized that increased abundance of pro-inflammatory cytokines in GM may be one of the mechanisms underlying the neurodegeneration of the GM cortex and the subsequent GM atrophy (Picon et al., 2021). In addition, varying levels of meningeal immune cell infiltration have been found in autopsies of patients with multiple sclerosis. In cases with increased levels of meningeal inflammation, broader demyelination along with neurosynaptic deficit in cortical GM were detected (Choi et al., 2012). Elevated levels of pro-inflammatory cytokines were detected in active lesions of MS, making immune mechanisms an intriguing and important aspect of GM injury research.

The present study is a retrospective research to identify diagnostic biomarkers associated with immune cell infiltration of GM lesions in MS by mining the GEO dataset. By analyzing the GM lesion expression profile data, a total of 502 DEGs were identified, including 265 up-regulated genes and 237 down-regulated genes. KEGG results indicated that the enriched pathways involved the MAPK signaling pathway, cAMP signaling pathway, and Toll-like receptor (TLR) signaling pathway. It has been demonstrated that excessive activation of MAPK in microglia of MS patients leads to microglia malfunction. Microglia cause local demyelination by impairing surrounding oligodendrocytes (Ten Bosch et al., 2021). cAMP is recognized as a potent negative regulator of T-cell immune function, and increased cAMP levels attenuate T-lymphocyte-mediated production of the pro-inflammatory cytokines IFN-γ and IL-1b. While reduced cAMP levels were observed in peripheral blood mononuclear cells of MS patients (Signorile et al., 2020). TLR ligands were identified as T-cell promoters in MS, and the expression of NF-κB DNA-binding activity was elevated in macrophages of MS patients after stimulation with TLR ligands, while upregulation of NF-κB-controlled adhesion molecules and cytokines could lead to an enhanced inflammatory response (Chen, Szodoray & Zeher, 2016). These results were substantially consistent with previous findings that the inflammatory response is involved in the pathogenesis of MS.

In this work, we identified that naive CD4^+^ T cells, activated NK cells, M0 macrophages, M1 macrophages and eosinophils were highly infiltrated in GM lesions. Genome-wide association studies and immunotherapy demonstrated that circulating CD4^+^ T cells are central players in the progression of MS. There are homeostatic changes in naive CD4^+^ T cells from MS patients. Such changes are associated with autoimmune disease. A recent study has extracted and sequenced naive CD4^+^ T cells from secondary progressive MS. The result suggested that changes in naive CD4 cell, particularly differences in T cell receptors and TLR signaling pathways, can determine the rate of disease progression in patients, which is critical for MS prognosis (Zastepa et al., 2014). In the research area of MS, NK cells have been basically neglected. With the recent expansion of knowledge on MS genetics, MS susceptibility loci were found to be significantly enriched in genes associated with cells of the innate immune system, including NK cells. In experimental autoimmune encephalomyelitis, NK cells are able to kill self-reactive T cells and inhibit Th17 differentiation and neurotrophic factor secretion in the CNS. NK cells are present in the CSF of MS patients and have a high cellular toxic activity against target stress cells. This suggests an additional regulatory role for NK cells in the development of MS, however, additional research is required to confirm that (Beliën, Goris & Matthys, 2022).

Presence of glial cells in GM injury indicates an important player in lesion formation. *In vivo* studies using PET have demonstrated presence of activated M1-type microglia in GM lesions in patients with MS. Like the CNS parenchymal macrophages, M1-type microglia secrete pro-inflammatory factors and cytotoxic factors, leading to demyelination and axonal injury, and may contribute to GM injury and increase clinical disability in MS patients (Jurga, Paleczna & Kuter, 2020). This is consistent with previous findings that the primary hallmark of inflammatory activity in GM pathology is significant activation of microglia, while microglia activation is linked to excessive infiltration of the meninges (Magliozzi et al., 2007). Microglia are thought to be a major influence on synaptic loss involved in MS because they remove synaptic connections to allow for appropriate brain connectivity during brain development (Tsouki & Williams, 2021). Microglia can also eliminate damaged synapses, which protects surrounding functionally normal synapses against damage from oxidative stress (Takahashi, Rochford & Neumann, 2005). Therefore, in GM injury, activation of microglia, while contributing to myelin loss and axonal injury, may in turn relate to the regression of inflammation and protection of synapses. Future more extensive studies on microglia may contribute to the advancement of GM injury research. Correlation analysis indicated that four genes were closely associated with immune cell infiltration, namely TLR9, CCL5, CXCL8 and PDGFRB. We combined these key indicators to establish a diagnosis nomogram. The calibration curve, DCA, and ROC curve confirmed that the prediction model had high accuracy for MS incidence risk.

TLR9 is localized in intracellular compartments and signals through MyD88 pathway, forming the MyD88-IRAK4 complex, leading to NF-κB activation, which in turn promotes the expression of various cytokines, including IFNβ and IL-10 (Zheng et al., 2019). Both IL-10 and IFNβ facilitate the anti-inflammatory environment of the CNS, and play a positive role in alleviating EAE (Dieu et al., 2021). In MS, IL-10 production was significantly reduced, possibly due to lower levels of TLR9 in B cells, indicating a potential immunomodulatory role for TLR9 in disease progression (Zhou et al., 2017).

Chemokines are key mediators in the immune surveillance process and can be secreted by activated T lymphocytes (Vilgelm & Richmond, 2019). By binding to chemokine receptors, chemokines activate intracellular signaling pathways associated with cell motility and activation (Chen et al., 2018), regulating the migration of macrophages and lymphocytes to CNS injury regions. The current study identified two chemokines as immune-related hub genes for GM injury: CCL5 and CXCL8. CCL5, also known as RANTES, is a marker of M1-type macrophages and highly expressed in active demyelinating lesions (An et al., 2021). In MS, astrocytes respond to IL17 and IL6 released by T cells and release large amounts of CCL5, which binds to CCR5 to control CNS monocyte/macrophage trafficking (Brambilla, 2019). Experimental studies have shown that CCL5 contributes to demyelination, meanwhile immune neutralizing antibodies against CCL5 inhibit the migration of inflammatory cells and CNS inflammatory infiltration, attenuating myelin loss in MS model animals (Cui, Chu & Chen, 2020). CXCL8 is a key mediator of inflammation-associated tissue damage and has no homologue in rodents (Russo et al., 2014). In the CNS, CXCL8 is produced by quiescent/activated endothelial cells and microglia (Hardeland, 2021). It recruits and activates leukocytes, regulating the expression of adhesion molecules in leukocytes and participating in the inflammatory response. CXCL8 is implicated in chemokine signaling and cytokine production, particularly the IL-17 signaling pathway, that may impact neutrophil recruitment and immunity. Chemotaxis of CXCL8 is mediated primarily through binding to its receptors CXCR1 and CXCR2 (Navarro et al., 2016). The effect of CXCL8 in MS is further reinforced due to its increased expression in peripheral blood (Bai et al., 2019). We observed decreased levels of CCL5 and CXCL8 in the MS group in GSE131282 dataset, however, *in vivo* experiment results showed that CCL5 protein and mRNA expressions were upregulated in the MS model. On one hand, due to the modulation of the chemokine system by various drugs in the treatment of MS, such as interferon-beta and methylprednisolone (Dhaiban et al., 2020), we speculate that the aberrant levels of CCL5 and CXCL8 in the dataset may be associated with MS patients being treated with related therapeutic strategies during their lifetime. It was shown that methylprednisolone application *in vitro* and *in vivo* increased the chemotaxis of monocytes from MS patients to CCL2, CCL5, and CX3CL1, and this chemotaxis may lead to abnormal levels of CCL5 (Fischer et al., 2019). In addition, IFN-β treatment decreases the expression of CCR5 on T cells from MS patients, and *in vitro* treatment of T cells with IFN-β inhibits the expression of this chemokine receptor and its ligands, including CCL5 and CCL3 (Zang et al., 2001). Treatment with rituximab also resulted in a significant decrease in the levels of CXCL8 and CXCL10 chemokines (de Flon et al., 2018). On the other hand, during CPZ-induced apoptosis of oligodendrocytes, astrocytes and microglia are activated, ultimately leading to demyelination of the brain (Zhan et al., 2020). While T cells and B cells exert a non-dominant action (Wolf et al., 2018), CPZ model fails to fully mimic all immune events in human MS brain, also leading to the discrepancy between the results in animal tissues and those in the dataset.

As a cell surface tyrosine kinase receptor, PDGFRB is capable of transducing extracellular signals into the cell, which is required for pericyte proliferation and migration (Nicolas et al., 2013; Xiang et al., 2019). Interestingly, pericytes are known to be in charge of the integrity of blood–brain barrier (BBB), and loss of BBB integrity and subsequent influx of inflammatory immune cells entering the CNS are characteristic of MS. Mice deficient in PDGFRB result in pericyte loss, BBB breakdown, damaged cerebrovascular coupling, and decreased capillary density (Miners, Schulz & Love, 2018). These lines of evidence suggested that aberrant PDGFRB expression is involved in the destruction of the BBB, causing infiltrative immune cells in the blood infiltration into the CNS, leading to MS neuroinflammation and disease progression.

The nomogram is a convenient tool for predicting the probability of a clinical event in an individual patient. Our nomogram was developed based on the four gene signature. To measure the accuracy and predictive performance of the nomogram prediction, we analyzed the Harrell C-index, calibration curve and DCA curve. The results showed that the nomogram has good applicability in predicting MS, which can help clinicians in decision making and has good clinical application.

In the current study, multiple pathways were identified to be associated with MS *via* GSEA analysis, such as cytokine-cytokine receptor interaction, JAK-STAT signaling pathway, cell adhesion molecules, *etc*. Activation of different types of immune cells produces multiple cytokines and has specific effects on cellular communication and interactions. Cytokines are released in a cascade, where one cytokine acts on target cells and produces other cytokines. In MS, antigen-presenting cells provide T cells with relevant antigens and produce a specific cytokine environment, and T cells use cytokines to recognize cell types, ultimately leading to complex cytokine-mediated communication signals (Gobel, Ruck & Meuth, 2018). The JAK/STAT signaling pathway is closely associated with many cytokines. Binding on cytokines and their receptors activates JAKs, which phosphorylate their primary substrate STAT and regulate target genes (Xin et al., 2020). A previous study has shown that JAK-STAT pathway gene expression is markedly altered during MS and overactivation of the JAK/STAT pathway has pathological significance in autoimmune diseases (Benveniste et al., 2014). Elevated levels of activated STAT3 are observed in T cells and monocytes of MS patients. Inhibition of the JAK/STAT3 pathway attenuates clinical symptoms, reduces immune cell infiltration in the CNS, and decreases pro-inflammatory cytokine and chemokine expression in animal models of MS (Liu et al., 2014). Cell adhesion molecules play a major regulatory role in the entry of immune cells into brain tissue. Elevated expression of adhesion molecules was detected in both cerebrovascular endothelial cells and inflammatory cells surrounding MS injury. It was shown that in MS, damage to the blood-brain barrier promotes the migration of T cells to the CNS, and activated T cells adhere to capillaries and interact with cell adhesion molecules to enter the CNS and promote the formation of demyelinating lesions (Mitosek-Szewczyk et al., 2010).

The present study still has some limitations. First, further functional impact of these hub genes in GM injury initiation and development needs to be evaluated at the animal level by gene knockout or overexpression experiments. Second, the original published dataset GSE135511 did not contain sufficient clinical information, such as therapeutic agents, resulting in the inability to make a reasonable analysis of the unusual expression of genes. In addition, the sample size included in the two datasets of our study remains small. Taking into account that the results may be biased by the small number of GEO samples we included and the sample deviation present in each dataset, this may lead to selectivity bias. Therefore, further validation in real-world data with a large sample size is necessary.

## Conclusions

In summary, this study identified a set of genes associated with GM injury in MS from the perspective of immune cell infiltration and developed a diagnosis model. These genes could contribute to understanding the mechanisms of MS and the effectively predicted diagnosis model could provide guidance for therapeutic intervention of MS.

## Supplemental Information

10.7717/peerj.15299/supp-1 Supplemental Information 1 Raw data.

10.7717/peerj.15299/supp-2 Supplemental Information 2 Differentially Expressed Genes identified in GSE135511.

10.7717/peerj.15299/supp-3 Supplemental Information 3 Immune-related genes obtained from InnateDB and ImmPort.

10.7717/peerj.15299/supp-4 Supplemental Information 4 Checklist.

## Additional Information and Declarations

### Data Availability

The following information was supplied regarding data availability: The raw data is available in the Supplemental Files. Publicly available datasets from GEO were analyzed in this study: GSE135511, GSE131282.

## References

[ref-1] An N, Yang J, Wang H, Sun S, Wu H, Li L, Li M (2021). Mechanism of mesenchymal stem cells in spinal cord injury repair through macrophage polarization. Cell & Bioscience.

[ref-2] Bai Z, Chen D, Wang L, Zhao Y, Liu T, Yu Y, Yan T, Cheng Y (2019). Cerebrospinal fluid and blood cytokines as biomarkers for multiple sclerosis: a systematic review and meta-analysis of 226 studies with 13,526 multiple sclerosis patients. Frontiers in Neuroscience.

[ref-3] Beliën J, Goris A, Matthys P (2022). Natural killer cells in multiple sclerosis: entering the stage. Frontiers in Immunology.

[ref-4] Benveniste EN, Liu Y, McFarland BC, Qin H (2014). Involvement of the janus kinase/signal transducer and activator of transcription signaling pathway in multiple sclerosis and the animal model of experimental autoimmune encephalomyelitis. Journal of Interferon & Cytokine Research.

[ref-5] Brambilla R (2019). The contribution of astrocytes to the neuroinflammatory response in multiple sclerosis and experimental autoimmune encephalomyelitis. Acta Neuropathologica.

[ref-6] Calabrese M, Magliozzi R, Ciccarelli O, Geurts JJ, Reynolds R, Martin R (2015). Exploring the origins of grey matter damage in multiple sclerosis. Nature Reviews Neuroscience.

[ref-7] Chen K, Bao Z, Tang P, Gong W, Yoshimura T, Wang JM (2018). Chemokines in homeostasis and diseases. Cellular & Molecular Immunology.

[ref-8] Chen JQ, Szodoray P, Zeher M (2016). Toll-like receptor pathways in autoimmune diseases. Clinical Reviews in Allergy & Immunology.

[ref-9] Choi SR, Howell OW, Carassiti D, Magliozzi R, Gveric D, Muraro PA, Nicholas R, Roncaroli F, Reynolds R (2012). Meningeal inflammation plays a role in the pathology of primary progressive multiple sclerosis. Brain.

[ref-10] Cui LY, Chu SF, Chen NH (2020). The role of chemokines and chemokine receptors in multiple sclerosis. International Immunopharmacology.

[ref-11] de Flon P, Soderstrom L, Laurell K, Dring A, Sundstrom P, Gunnarsson M, Svenningsson A (2018). Immunological profile in cerebrospinal fluid of patients with multiple sclerosis after treatment switch to rituximab and compared with healthy controls. PLOS ONE.

[ref-12] Dhaiban S, Al-Ani M, Elemam NM, Maghazachi AA (2020). Targeting chemokines and chemokine receptors in multiple sclerosis and experimental autoimmune encephalomyelitis. Journal of Inflammation Research.

[ref-13] Dieu RS, Wais V, Sorensen MZ, Marczynska J, Dubik M, Kavan S, Thomassen M, Burton M, Kruse T, Khorooshi R, Owens T (2021). Central nervous system-endogenous TLR7 and TLR9 induce different immune responses and effects on experimental autoimmune encephalomyelitis. Frontiers in Neuroscience.

[ref-14] Eshaghi A, Marinescu RV, Young AL, Firth NC, Prados F, Jorge Cardoso M, Tur C, De Angelis F, Cawley N, Brownlee WJ, De Stefano N, Laura Stromillo M, Battaglini M, Ruggieri S, Gasperini C, Filippi M, Rocca MA, Rovira A, Sastre-Garriga J, Geurts JJG, Vrenken H, Wottschel V, Leurs CE, Uitdehaag B, Pirpamer L, Enzinger C, Ourselin S, Gandini Wheeler-Kingshott CA, Chard D, Thompson AJ, Barkhof F, Alexander DC, Ciccarelli O (2018). Progression of regional grey matter atrophy in multiple sclerosis. Brain.

[ref-15] Fischer HJ, Finck TLK, Pellkofer HL, Reichardt HM, Luhder F (2019). Glucocorticoid therapy of multiple sclerosis patients induces anti-inflammatory polarization and increased chemotaxis of monocytes. Frontiers in Immunology.

[ref-16] Gelfand JM, Cree BAC, Hauser SL (2017). Ocrelizumab and other CD20(+) B-cell-depleting therapies in multiple sclerosis. Neurotherapeutics.

[ref-17] Gobel K, Ruck T, Meuth SG (2018). Cytokine signaling in multiple sclerosis: lost in translation. Multiple Sclerosis Journal.

[ref-18] Hardeland R (2021). Melatonin and microglia. International Journal of Molecular Sciences.

[ref-19] Hnilicova P, Strbak O, Kolisek M, Kurca E, Zelenak K, Sivak S, Kantorova E (2020). Current methods of magnetic resonance for noninvasive assessment of molecular aspects of pathoetiology in multiple sclerosis. International Journal of Molecular Sciences.

[ref-20] Jurga AM, Paleczna M, Kuter KZ (2020). Overview of general and discriminating markers of differential microglia phenotypes. Frontiers in Cellular Neuroscience.

[ref-21] Kawachi I (2014). Deep grey matter involvement in multiple sclerosis: key player or bystander?. Journal of Neurology, Neurosurgery & Psychiatry.

[ref-22] Langfelder P, Horvath S (2008). WGCNA: an R package for weighted correlation network analysis. BMC Bioinformatics.

[ref-23] Lassmann H (2018). Pathogenic mechanisms associated with different clinical courses of multiple sclerosis. Frontiers in Immunology.

[ref-24] Liu Y, Holdbrooks AT, De Sarno P, Rowse AL, Yanagisawa LL, McFarland BC, Harrington LE, Raman C, Sabbaj S, Benveniste EN, Qin H (2014). Therapeutic efficacy of suppressing the Jak/STAT pathway in multiple models of experimental autoimmune encephalomyelitis. Journal of Immunology.

[ref-25] Magliozzi R, Howell OW, Durrenberger P, Arico E, James R, Cruciani C, Reeves C, Roncaroli F, Nicholas R, Reynolds R (2019). Meningeal inflammation changes the balance of TNF signalling in cortical grey matter in multiple sclerosis. Journal of Neuroinflammation.

[ref-26] Magliozzi R, Howell O, Vora A, Serafini B, Nicholas R, Puopolo M, Reynolds R, Aloisi F (2007). Meningeal B-cell follicles in secondary progressive multiple sclerosis associate with early onset of disease and severe cortical pathology. Brain.

[ref-27] Magliozzi R, Reynolds R, Calabrese M (2018). MRI of cortical lesions and its use in studying their role in MS pathogenesis and disease course. Brain Pathology.

[ref-28] Miners JS, Schulz I, Love S (2018). Differing associations between Aβ accumulation, hypoperfusion, blood-brain barrier dysfunction and loss of PDGFRB pericyte marker in the precuneus and parietal white matter in Alzheimer’s disease. Journal of Cerebral Blood Flow & Metabolism.

[ref-29] Mitosek-Szewczyk K, Stelmasiak Z, Bartosik-Psujek H, Belniak E (2010). Impact of cladribine on soluble adhesion molecules in multiple sclerosis. Acta Neurologica Scandinavica.

[ref-30] Navarro R, Compte M, Alvarez-Vallina L, Sanz L (2016). Immune regulation by pericytes: modulating innate and adaptive immunity. Frontiers in Immunology.

[ref-31] Newman AM, Liu CL, Green MR, Gentles AJ, Feng W, Xu Y, Hoang CD, Diehn M, Alizadeh AA (2015). Robust enumeration of cell subsets from tissue expression profiles. Nature Methods.

[ref-32] Nicolas G, Pottier C, Maltête D, Coutant S, Rovelet-Lecrux A, Legallic S, Rousseau S, Vaschalde Y, Guyant-Maréchal L, Augustin JJN (2013). Mutation of the PDGFRB gene as a cause of idiopathic basal ganglia calcification. Neurology.

[ref-33] Picon C, Jayaraman A, James R, Beck C, Gallego P, Witte ME, van Horssen J, Mazarakis ND, Reynolds R (2021). Neuron-specific activation of necroptosis signaling in multiple sclerosis cortical grey matter. Acta Neuropathologica.

[ref-34] Reali C, Magliozzi R, Roncaroli F, Nicholas R, Howell OW, Reynolds R (2020). B cell rich meningeal inflammation associates with increased spinal cord pathology in multiple sclerosis. Brain Pathology.

[ref-35] Ritchie ME, Phipson B, Wu D, Hu Y, Law CW, Shi W, Smyth GK (2015). limma powers differential expression analyses for RNA-sequencing and microarray studies. Nucleic Acids Research.

[ref-36] Russo RC, Garcia CC, Teixeira MM, Amaral FA (2014). The CXCL8/IL-8 chemokine family and its receptors in inflammatory diseases. Expert Review of Clinical Immunology.

[ref-37] Signorile A, Ferretta A, Ruggieri M, Paolicelli D, Lattanzio P, Trojano M, De Rasmo D (2020). Mitochondria, oxidative stress, cAMP signalling and apoptosis: a crossroads in lymphocytes of multiple sclerosis, a possible role of nutraceutics. Antioxidants (Basel).

[ref-38] Takahashi K, Rochford CD, Neumann H (2005). Clearance of apoptotic neurons without inflammation by microglial triggering receptor expressed on myeloid cells-2. Journal of Experimental Medicine.

[ref-39] Ten Bosch GJA, Bolk J, t Hart BA, Laman JD (2021). Multiple sclerosis is linked to MAPK(ERK) overactivity in microglia. Journal of Molecular Medicine.

[ref-40] Tremlett H, Marrie RA (2021). The multiple sclerosis prodrome: emerging evidence, challenges, and opportunities. Multiple Sclerosis Journal.

[ref-41] Tsouki F, Williams A (2021). Multifaceted involvement of microglia in gray matter pathology in multiple sclerosis. Stem Cells (Dayton, Ohio).

[ref-42] van Langelaar J, Rijvers L, Smolders J, van Luijn MM (2020). B and T cells driving multiple sclerosis: identity, mechanisms and potential triggers. Frontiers in Immunology.

[ref-43] Vercellino M, Marasciulo S, Grifoni S, Vallino-Costassa E, Bosa C, Pasanisi MB, Crociara P, Casalone C, Chio A, Giordana MT, Corona C, Cavalla P (2022). Acute and chronic synaptic pathology in multiple sclerosis gray matter. Multiple Sclerosis.

[ref-44] Vilgelm AE, Richmond A (2019). Chemokines modulate immune surveillance in tumorigenesis, metastasis, and response to immunotherapy. Frontiers in Immunology.

[ref-45] Wolf Y, Shemer A, Levy-Efrati L, Gross M, Kim JS, Engel A, David E, Chappell-Maor L, Grozovski J, Rotkopf R, Biton I, Eilam-Altstadter R, Jung S (2018). Microglial MHC class II is dispensable for experimental autoimmune encephalomyelitis and cuprizone-induced demyelination. European Journal of Immunology.

[ref-46] Xiang DN, Feng YF, Wang J, Zhang X, Shen JJ, Zou R, Yuan YZ (2019). Platelet-derived growth factor-BB promotes proliferation and migration of retinal microvascular pericytes by up-regulating the expression of C-X-C chemokine receptor types 4. Experimental and Therapeutic Medicine.

[ref-47] Xin P, Xu X, Deng C, Liu S, Wang Y, Zhou X, Ma H, Wei D, Sun S (2020). The role of JAK/STAT signaling pathway and its inhibitors in diseases. International Immunopharmacology.

[ref-48] Yu G, Wang L, Han Y, He Q (2012). clusterProfiler: an R package for comparing biological themes among gene clusters. OMICS: A Journal of Integrative Biology.

[ref-49] Zang YC, Halder JB, Samanta AK, Hong J, Rivera VM, Zhang JZ (2001). Regulation of chemokine receptor CCR5 and production of RANTES and MIP-1alpha by interferon-beta. Journal of Neuroimmunology.

[ref-50] Zastepa E, Fitz-Gerald L, Hallett M, Antel J, Bar-Or A, Baranzini S, Lapierre Y, Haegert DG (2014). Naive CD4 T-cell activation identifies MS patients having rapid transition to progressive MS. Neurology.

[ref-51] Zhan J, Mann T, Joost S, Behrangi N, Frank M, Kipp M (2020). The cuprizone model: dos and do nots. Cells.

[ref-52] Zheng C, Chen J, Chu F, Zhu J, Jin T (2019). Inflammatory role of TLR-MyD88 signaling in multiple sclerosis. Frontiers in Molecular Neuroscience.

[ref-53] Zhou Y, Fang L, Peng L, Qiu W (2017). TLR9 and its signaling pathway in multiple sclerosis. Journal of the Neurological Sciences.

