# Supplementary figures and images for: Discovery of grey matter lesion-related immune genes for diagnostic prediction in multiple sclerosis
Source: PeerJ. 2023 Apr 26;11:e15299. doi: 10.7717/peerj.15299 (PMC10148642; doi:10.7717/peerj.15299)

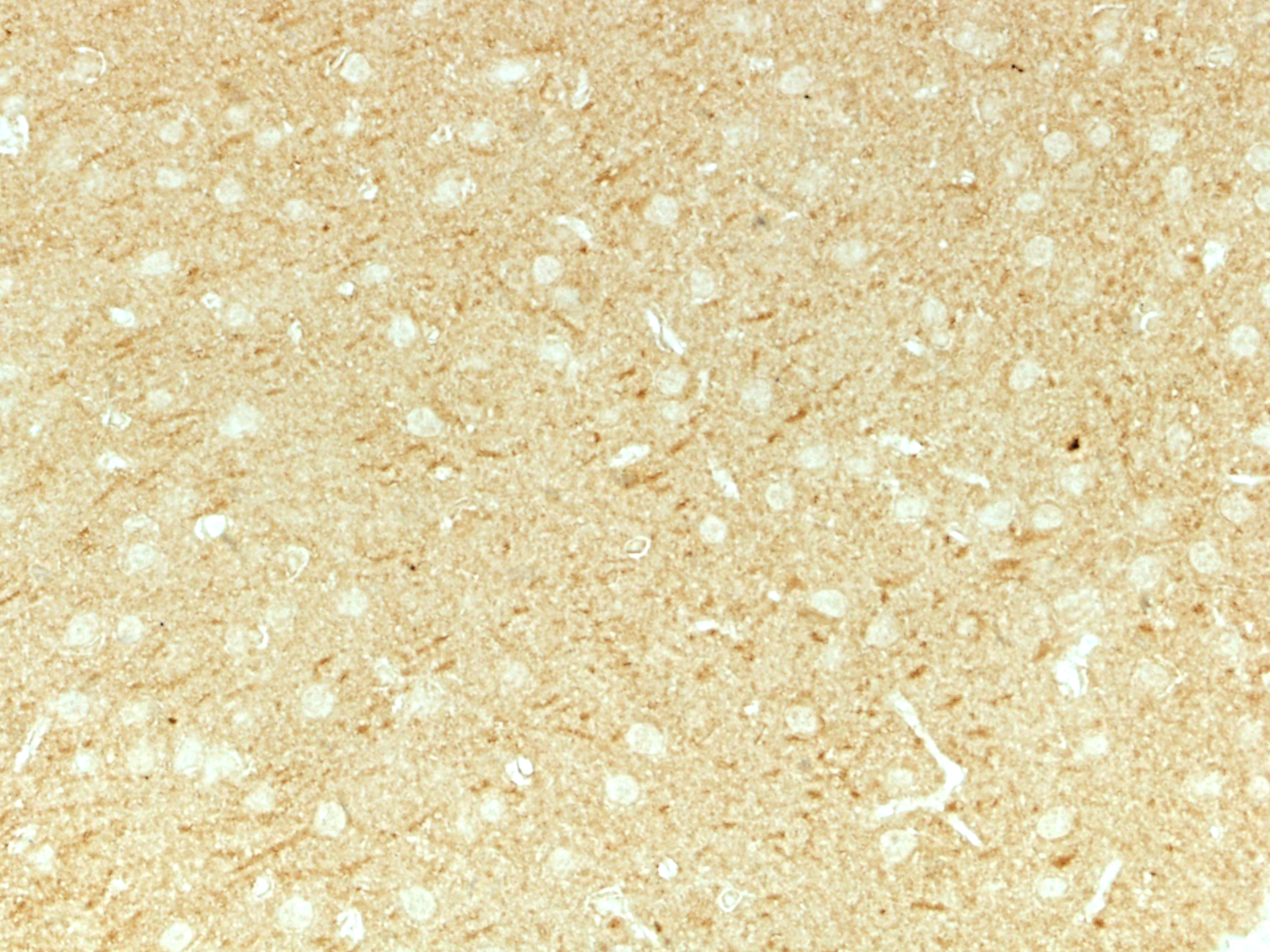

Supplement: Supplemental Information 1 [file peerj-11-15299-s001.zip › raw data/IHC/cpz-CCL5-40x.tif]

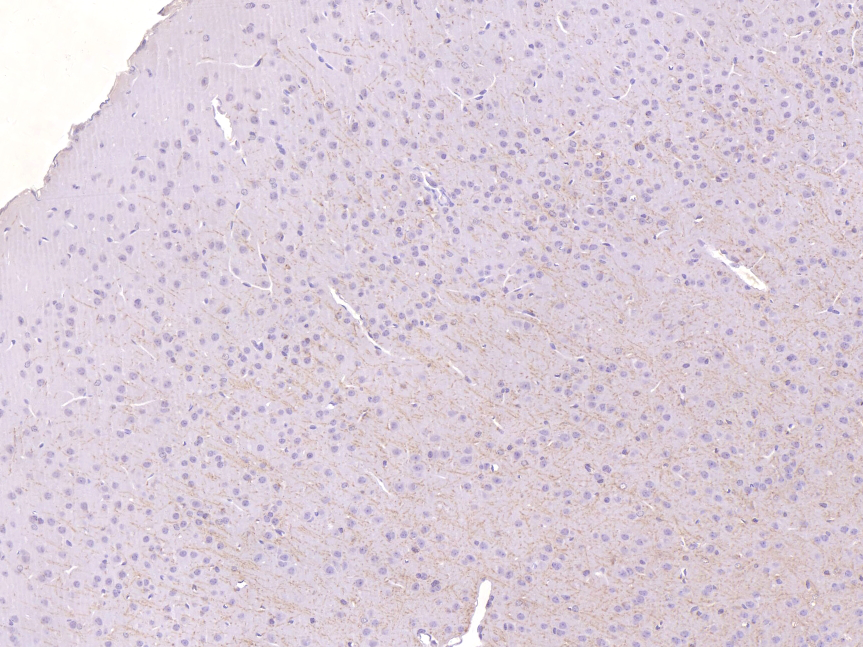

Supplement: Supplemental Information 1 [file peerj-11-15299-s001.zip › raw data/IHC/cpz-MBP-10x.tif]

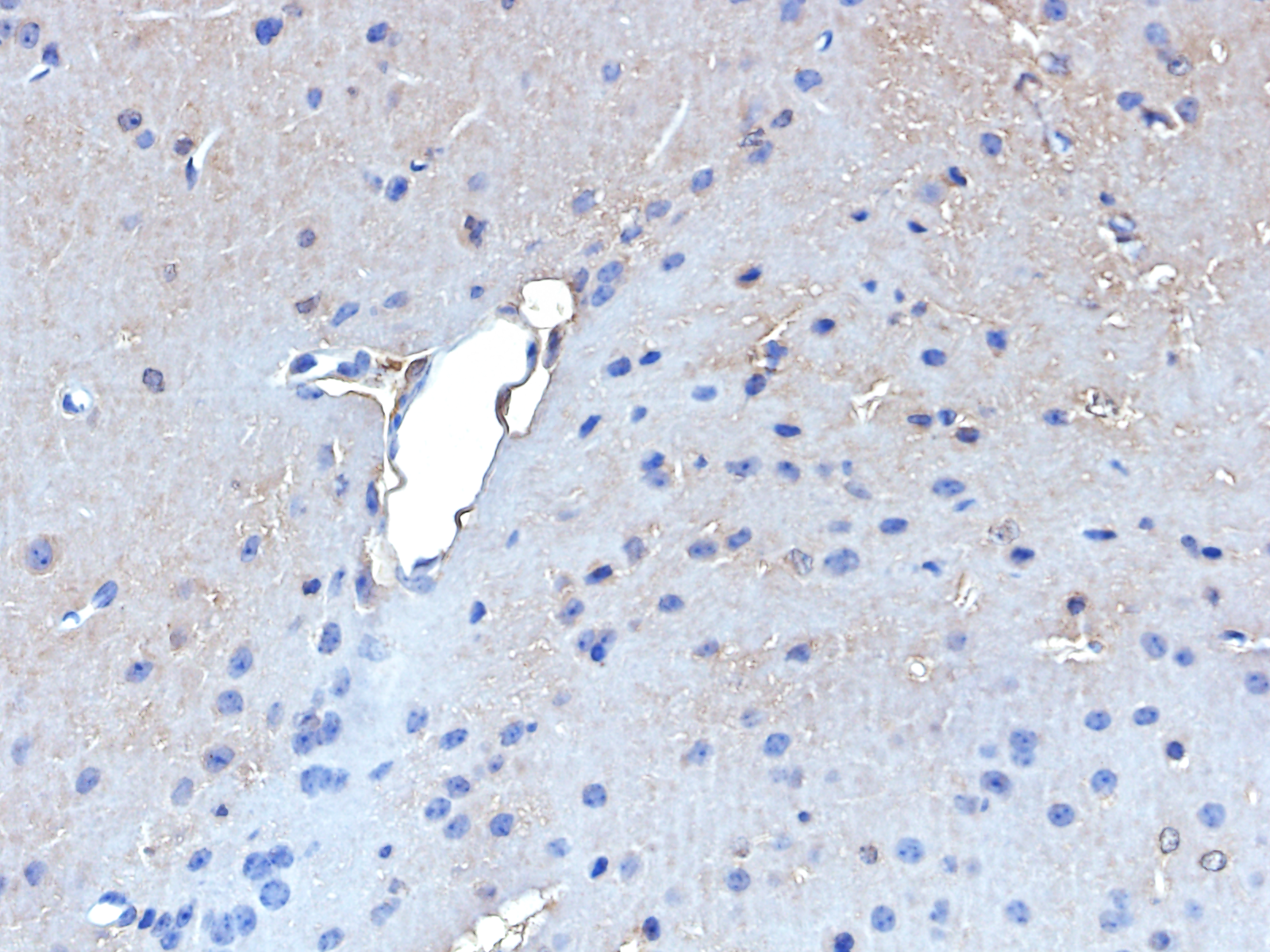

Supplement: Supplemental Information 1 [file peerj-11-15299-s001.zip › raw data/IHC/cpz-PDGFRB-40x.tif]

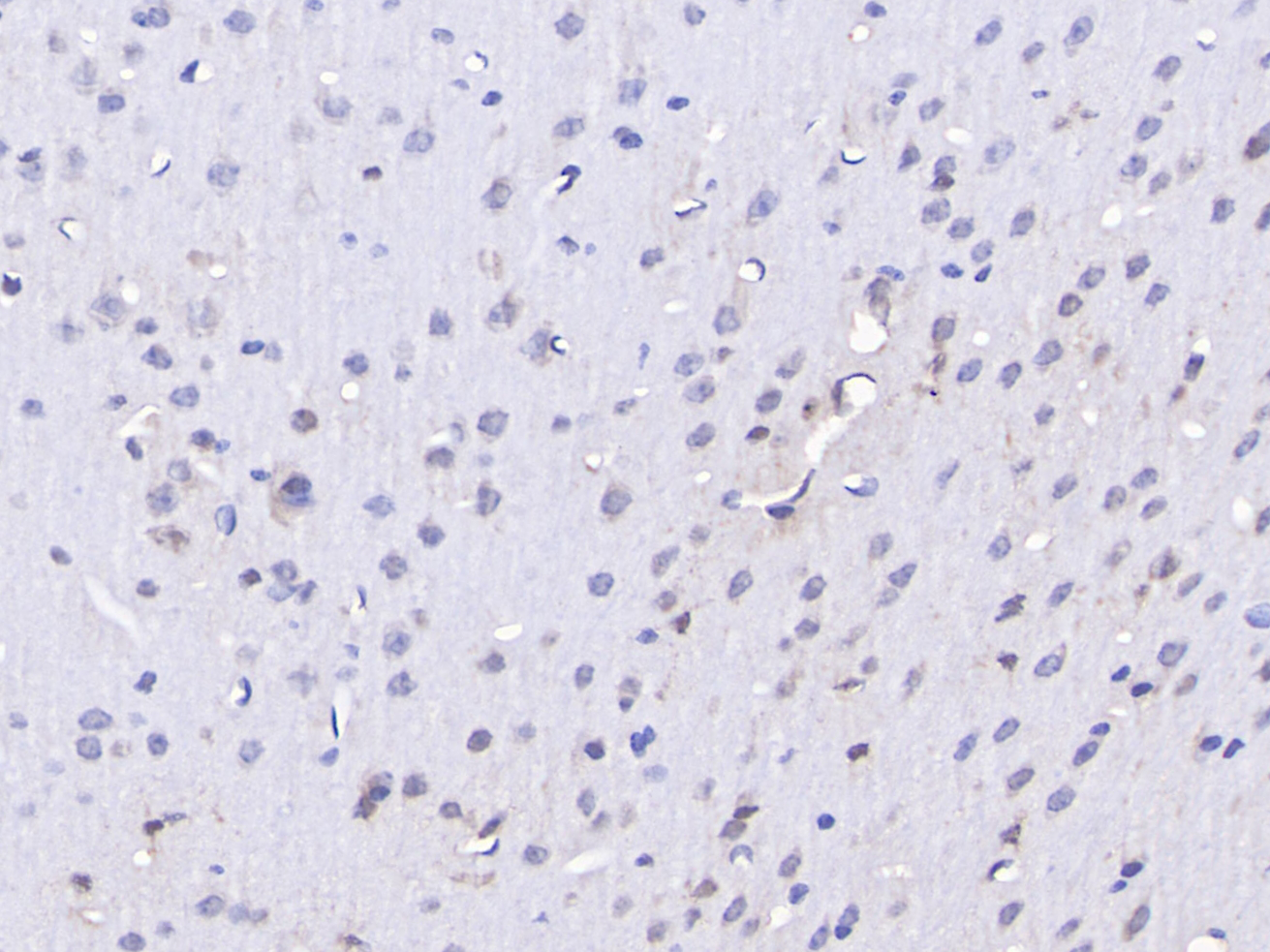

Supplement: Supplemental Information 1 [file peerj-11-15299-s001.zip › raw data/IHC/cpz-TLR9-40x.tif]

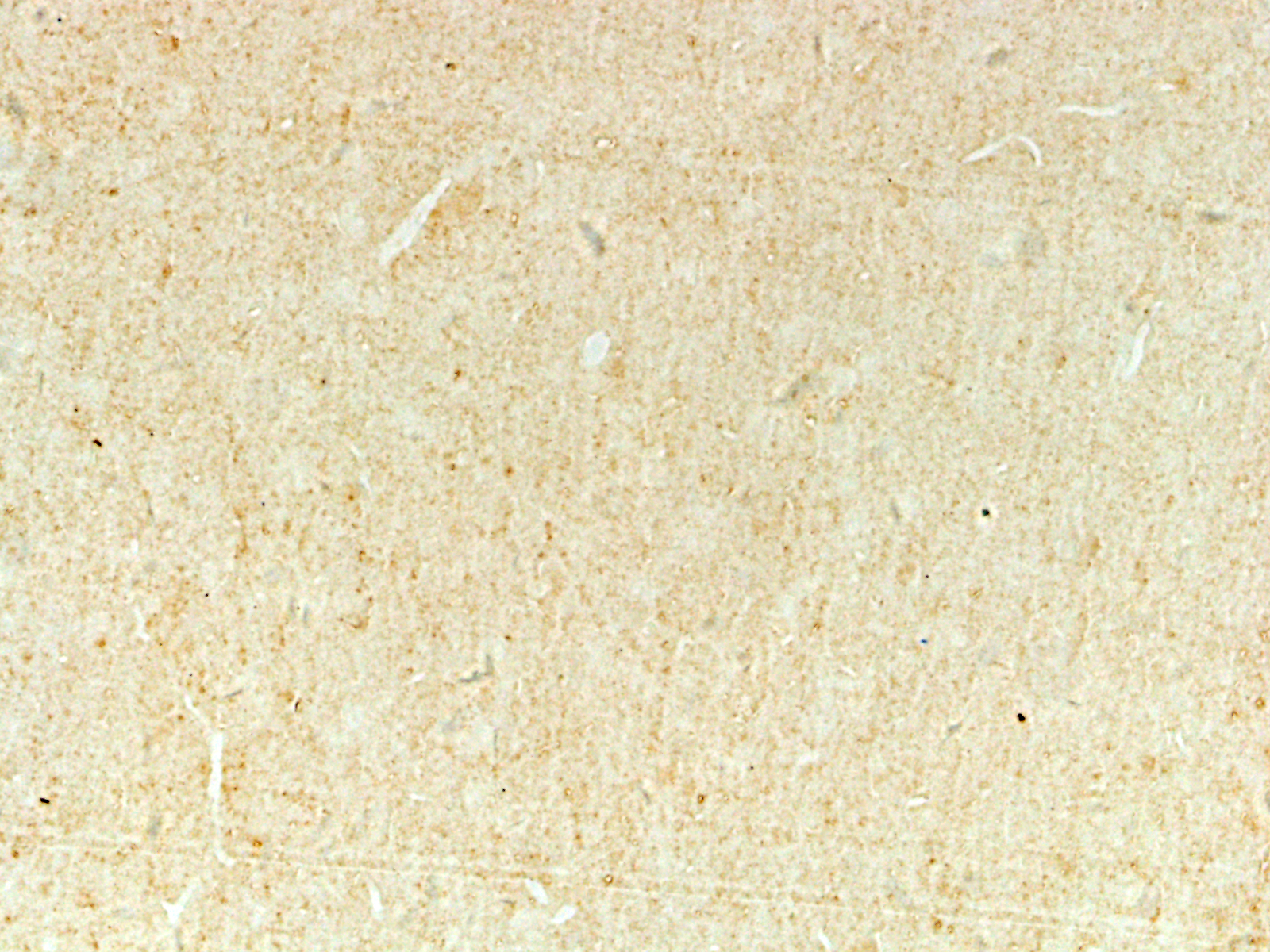

Supplement: Supplemental Information 1 [file peerj-11-15299-s001.zip › raw data/IHC/nc-CCL5-40x.tif]

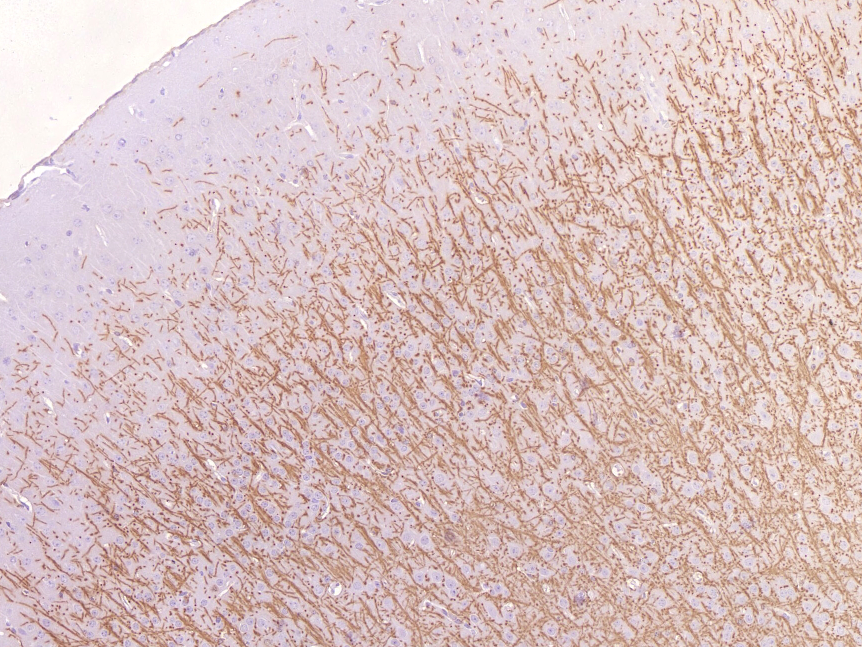

Supplement: Supplemental Information 1 [file peerj-11-15299-s001.zip › raw data/IHC/nc-MBP-10x.tif]

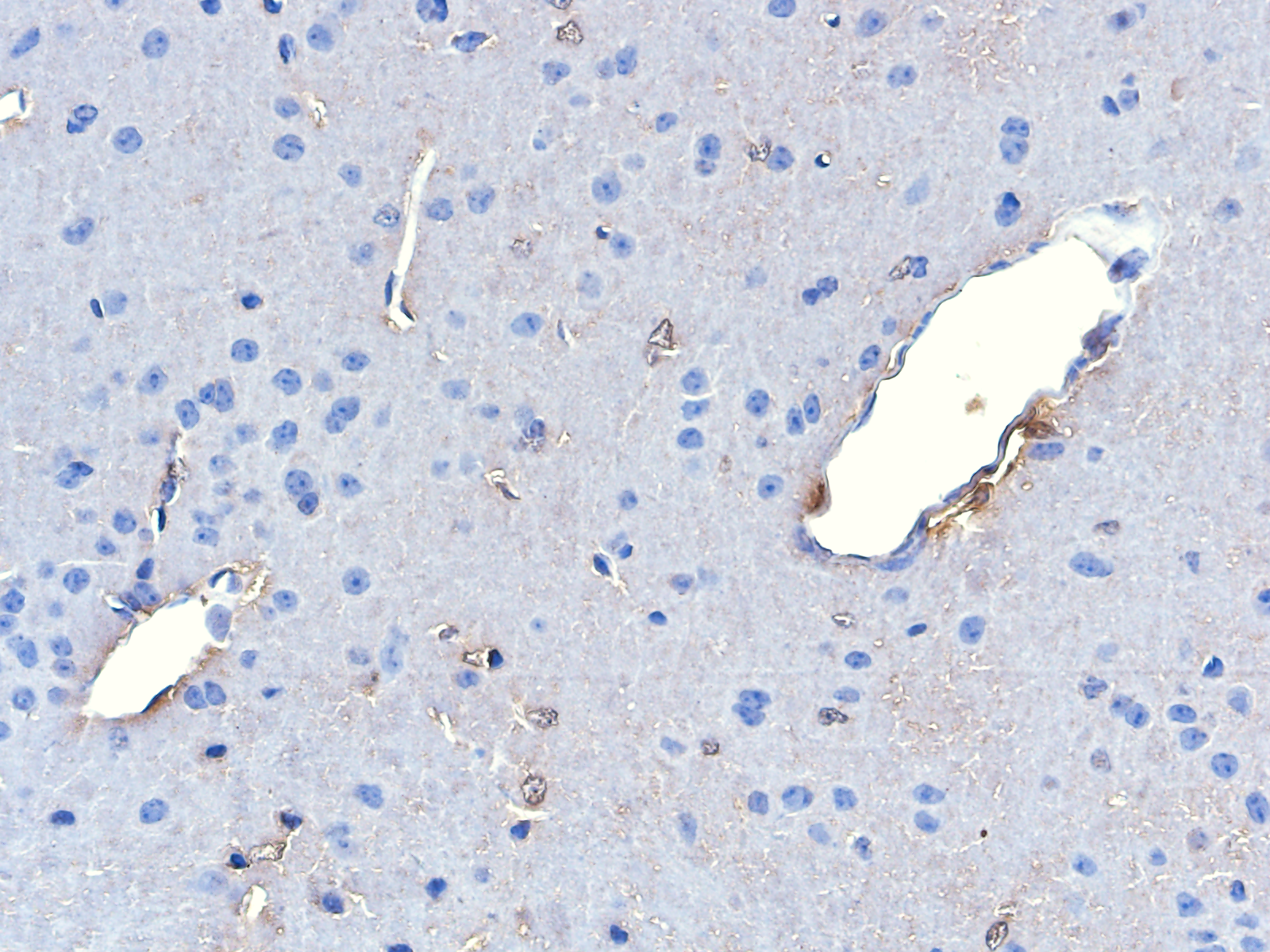

Supplement: Supplemental Information 1 [file peerj-11-15299-s001.zip › raw data/IHC/nc-PDGFRB-40x.tif]

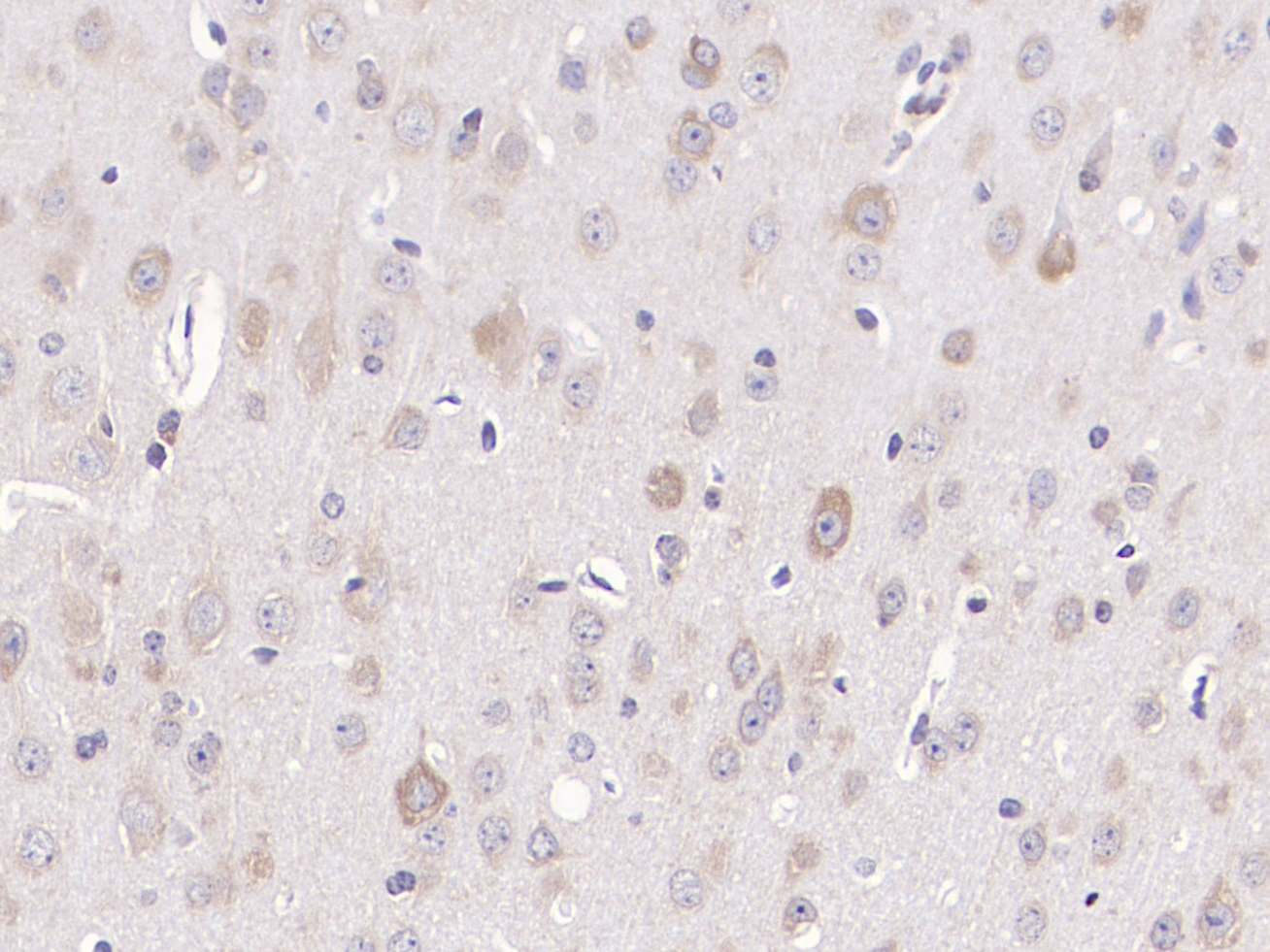

Supplement: Supplemental Information 1 [file peerj-11-15299-s001.zip › raw data/IHC/nc-TLR9-40x.tif]

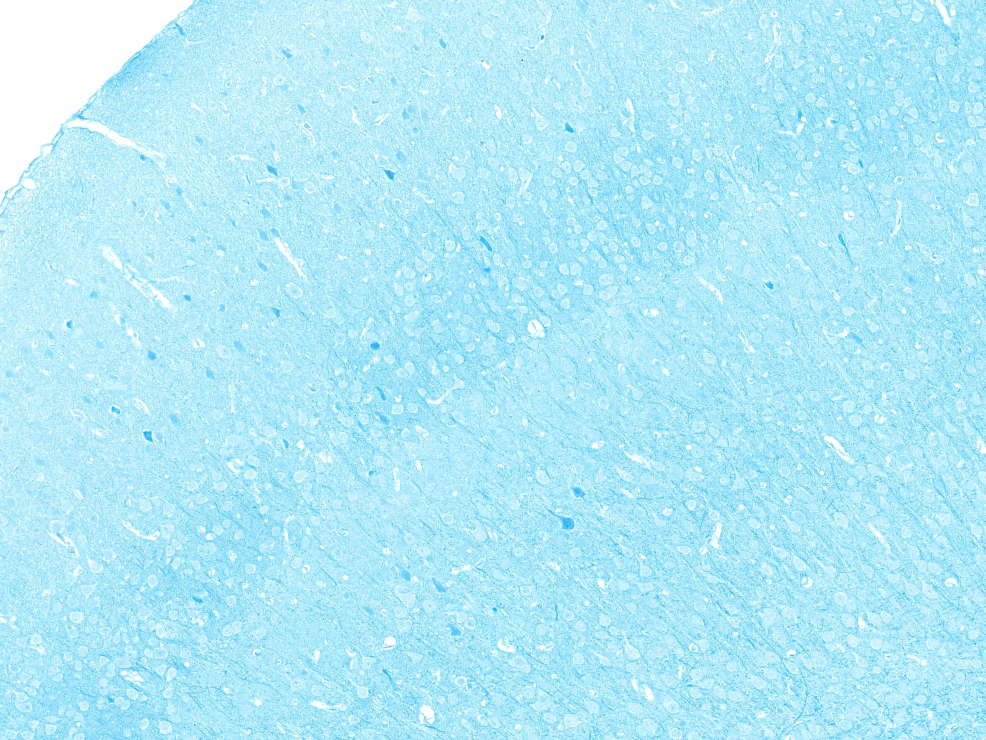

Supplement: Supplemental Information 1 [file peerj-11-15299-s001.zip › raw data/LFB/cpz-LFB-10x.tif]

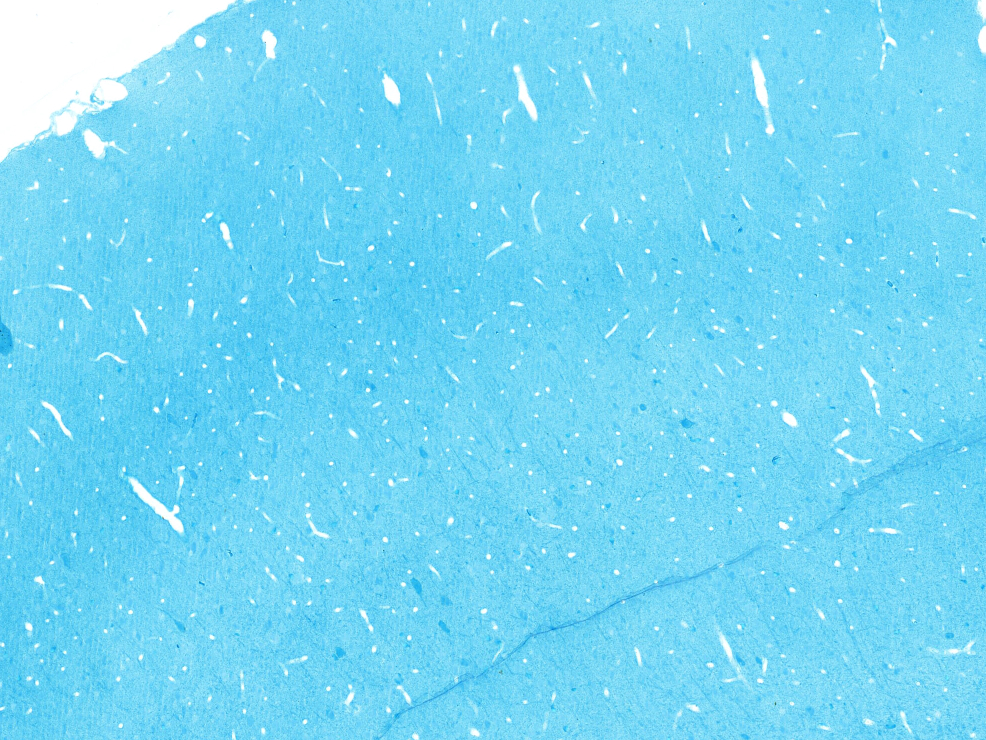

Supplement: Supplemental Information 1 [file peerj-11-15299-s001.zip › raw data/LFB/nc-LFB-10x.tif]

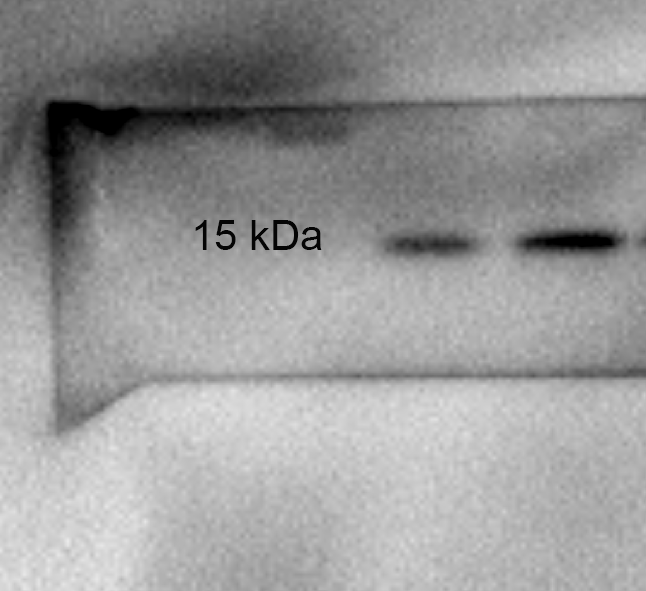

Supplement: Supplemental Information 1 [file peerj-11-15299-s001.zip › raw data/WB/ccl5.tif]

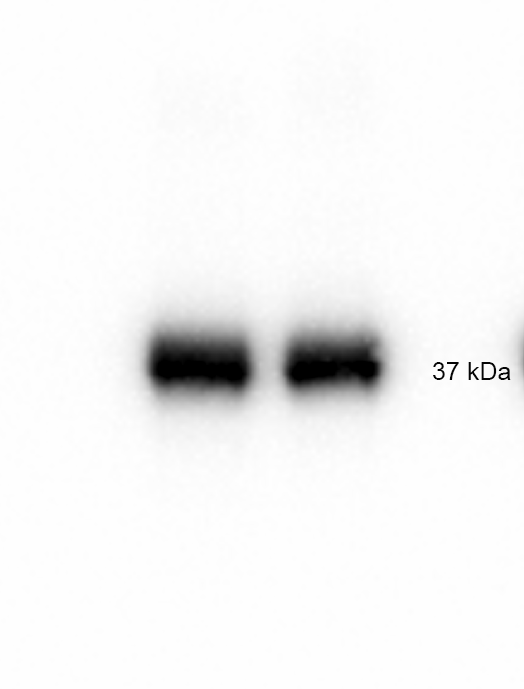

Supplement: Supplemental Information 1 [file peerj-11-15299-s001.zip › raw data/WB/GAPDH .tif]

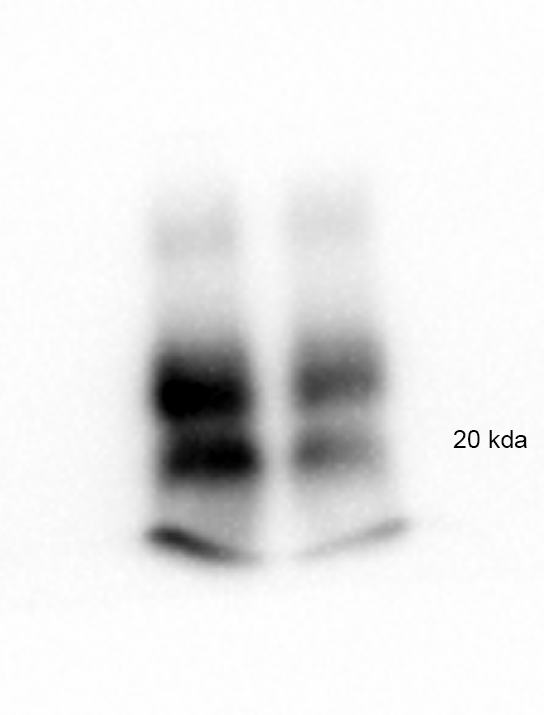

Supplement: Supplemental Information 1 [file peerj-11-15299-s001.zip › raw data/WB/MBP.tif]

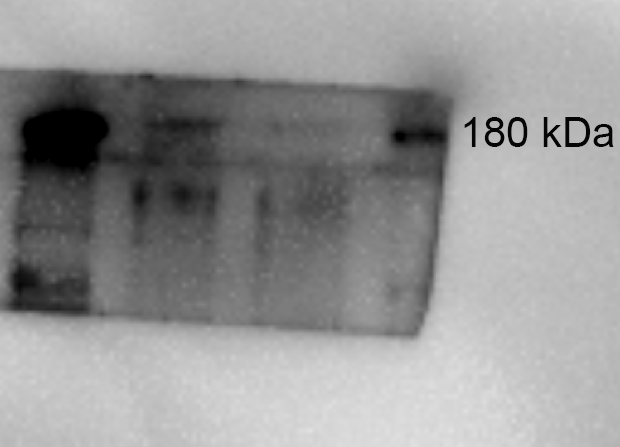

Supplement: Supplemental Information 1 [file peerj-11-15299-s001.zip › raw data/WB/PDGFRB.tif]

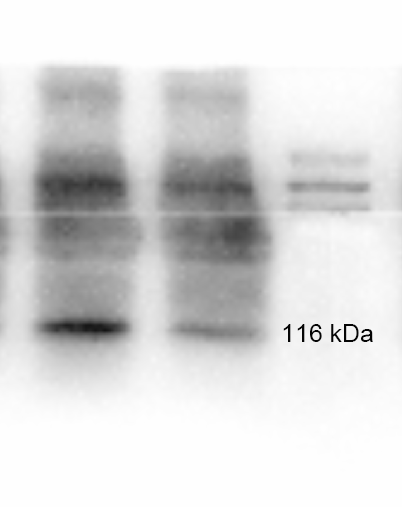

Supplement: Supplemental Information 1 [file peerj-11-15299-s001.zip › raw data/WB/tlr9.tif]
